# Mechanistic basis of Nek7 activation through Nek9 binding and induced dimerization

**DOI:** 10.1038/ncomms9771

**Published:** 2015-11-02

**Authors:** Tamanna Haq, Mark W. Richards, Selena G. Burgess, Pablo Gallego, Sharon Yeoh, Laura O’Regan, David Reverter, Joan Roig, Andrew M. Fry, Richard Bayliss

**Affiliations:** 1 Department of Biochemistry, University of Leicester, Lancaster Road, Leicester LE1 9HN, UK; 2 Cancer Research UK Leicester Centre, Leicester LE1 9HN, UK; 3 Departament de Bioquimica i Biologia Molecular, Institut de Biotecnologia i de Biomedicina, Universitat Autònoma de Barcelona, 08193 Bellaterra, Spain; 4 Cell and Developmental Biology Program, Institute for Research in Biomedicine, 08028 Barcelona, Spain

## Abstract

Mitotic spindle assembly requires the regulated activities of protein kinases such as Nek7 and Nek9. Nek7 is autoinhibited by the protrusion of Tyr97 into the active site and activated by the Nek9 non-catalytic C-terminal domain (CTD). CTD binding apparently releases autoinhibition because mutation of Tyr97 to phenylalanine increases Nek7 activity independently of Nek9. Here we find that self-association of the Nek9-CTD is needed for Nek7 activation. We map the minimal Nek7 binding region of Nek9 to residues 810–828. A crystal structure of Nek7^Y97F^ bound to Nek9^810–828^ reveals a binding site on the C-lobe of the Nek7 kinase domain. Nek7^Y97F^ crystallizes as a back-to-back dimer between kinase domain N-lobes, in which the specific contacts within the interface are coupled to the conformation of residue 97. Hence, we propose that the Nek9-CTD activates Nek7 through promoting back-to-back dimerization that releases the autoinhibitory tyrosine residue, a mechanism conserved in unrelated kinase families.

## Abstract (web summary)

NEK7, a kinase involved in mitosis, is regulated by the kinase NEK9. Here the authors identify the region in NEK9 that binds NEK7 and find that the mechanism of regulation involves dimerization coupled to structural changes in the active site.

## Graphical abstract

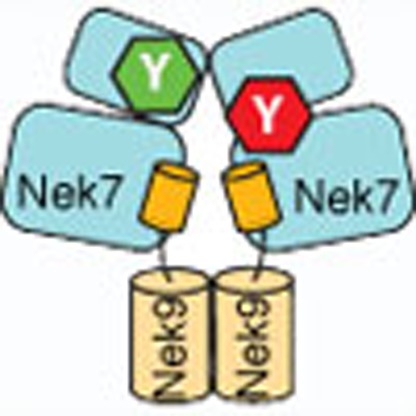

Protein kinases are regulated through conformational changes in the catalytic domain brought about through phosphorylation, self-association or the binding of protein partners^1^,^2^,^3^,^4^. Many protein kinases are activated through the phosphorylation of Ser, Thr or Tyr residues within the activation loop, which is stabilized to facilitate the binding of protein substrates^5^,^6^. This phosphorylation event can be catalysed by an upstream kinase, as happens in signalling cascades, or can be an autocatalytic event, as happens in receptor tyrosine kinases on dimerization.

Many kinases can exist in an autoinhibited conformation that is incompatible with catalytic activity and are converted to an active conformation through a regulated mechanism. In one such mechanism, first identified in CDKs and Src-family kinases, the activation loop forms a short α-helix that stabilizes the αC-helix in an outward position relative to what is observed in active kinase structures^7^,^8^,^9^,^10^. This outward position of the αC-helix disrupts a conserved salt bridge formed between a glutamic acid on the αC-helix and a lysine on the β3-strand that positions the γ-phosphate of ATP for catalysis. Another hallmark of an inactive kinase evident in the CDK/Src-like autoinhibited conformation is a disrupted regulatory- or R-spine, which is made up of the side chains of four hydrophobic residues: the first residue of the β4 strand in the N-terminal lobe, a residue in the final turn of the αC-helix, the middle residue of the DFG motif and the first residue of the HRD motif^11^,^12^. These elements of the CDK/Src-like autoinhibited conformation are conserved among many kinases whose regulatory mechanisms are otherwise quite distinct. This example is motivating researchers to look for other unifying themes in kinase regulation that will help to simplify and make sense of the extensive kinomes of humans and other organisms.

Another common theme is protein kinase activation through self-association. This process is governed by a number of distinct structural mechanisms, which have recently been comprehensively reviewed^4^. The eIF4α and IRE1/RNAseL protein kinase families provide an interesting example of activation through dimerization. Crystal structures of active, dimeric forms of these kinases reveal back-to-back dimers in which the active sites of each protomer face outwards. These dimers are formed by a symmetric association of kinase catalytic domains through an interface involving primarily their N-terminal lobes^13^,^14^,^15^,^16^,^17^. Crystal structures of inactive forms of these kinases, where available, show disrupted R-spines and a shifted and/or unwound αC-helix^18^,^19^. In these kinases, dimerization promotes kinase activation by formation of a back-to-back interface that is coupled to the formation of an active conformation^4^.

The 11 members of the human NIMA-related Ser/Thr kinase (Nek) family have cell cycle-related signalling functions during mitosis, on DNA damage and in cilia^20^. Nek6, Nek7 and Nek9 contribute to mitotic spindle assembly during early mitosis with the microtubule motor protein Eg5 and heat shock protein Hsp72 as substrates of Nek6, and the centrosome-associated NEDD1 protein a substrate of Nek9 (refs 21, 22, 23, 24, 25). Nek6 and Nek7 are also substrates of Nek9 that are activated through phosphorylation of key residues within their activation loops^26^,^27^. Nek6 and Nek7 are closely related proteins that comprises a highly conserved catalytic domain preceded by a short divergent N-terminal extension (NTE) that determines their distinct substrate preference^28^. Nek9 has an N-terminal kinase domain followed by an RCC1-like β-propeller domain and a C-terminal domain (CTD) comprising an unstructured region and a putative coiled-coil (CC). Nek6 and Nek7 interact with Nek9 through a region in the CTD that lies N terminal to the CC and this interaction is blocked in interphase by cell cycle-dependent association of Nek9 with LC8 (refs 29, 30). The non-catalytic CTD of Nek9 provides a second and alternative mechanism of Nek6 and Nek7 activation through binding and allosteric relief of an autoinhibited state^31^.

Mechanistic insights into these kinases have been gained through the structures of Nek9 aa 940–949 bound to LC8 (ref. 29) and the structure of full-length Nek7 in an unphosphorylated, inactive form^31^. The structure of Nek7 revealed an autoinhibited conformation characterized by the orientation of Tyr97 with the side chain pointing into the active site, stabilizing the αC-helix in an outwards position. Mutation of Nek7 Tyr97 to Ala, Leu or Phe results in a more active kinase, consistent with an autoinhibitory role of Tyr97. Nek9-CTD increases the activity of wild-type (WT) Nek7 to that of the Y97A mutant alone, suggesting this autoinhibitory mechanism is relieved through binding of Nek9-CTD. However, the mechanism through which the Nek9-CTD stimulates Nek7 catalytic activity is unknown. It also remains to be discovered exactly how Tyr97 mutations increase kinase activity, and how this relates to the mechanism of activation by Nek9-CTD. In this study, we address these questions through biochemical approaches interpreted in the light of the structure of Nek7^Y97F^ bound to a fragment of Nek9-CTD. We find that the activation of Nek7 by Nek9-CTD unexpectedly resembles the mechanism that governs the activation of kinases from the eIF4α and IRE1 families.

## Results

### Nek9 810–828 is the minimal Nek7 binding region

We set out to define the minimal region of the Nek9-CTD (aa 726–979) that was still capable of binding to Nek7. Fragments of Nek9-CTD truncated at the N terminus or C terminus were generated as glutathione S-transferase (GST) tagged fusions. Nek9–Nek7 binding was visualized through immunoblotting with an anti-His_6_ antibody to detect bound Nek7-His_6_ (Supplementary Fig. 1a). The smallest fragment that retained Nek7 binding comprised the residues 810–828 that lies towards the middle of the unstructured region of the Nek9-CTD.

The next experiments investigated whether this region is necessary for Nek7 binding in the context of the Nek9-CTD using point mutations in and surrounding this region (Supplementary Fig. 1b,c). We first focused on a subset of aromatic or hydrophobic residues sharing high sequence conservation with other Nek9 homologues (Supplementary Fig. 2). Nek9^L812A^, Nek9^L816A^, Nek9^F821A^ and Nek9^I822A^ ablated binding to Nek7 (Supplementary Fig. 1b), whereas mutations outside the 810–828 region had no effect, thus confirming that this region is necessary and sufficient for Nek9-CTD binding to Nek7.

Next, alanine-scanning mutagenesis through the minimal binding region was performed to pinpoint the contribution of each residue to the interaction. Several amino acids immediately N terminal to the mapped region were included, as this region is also highly conserved between Nek9 homologues (Supplementary Fig. 2). Mutations Nek9^P809A^ and Nek9^W811A^ ablated binding to Nek7 suggesting these residues form key stabilizing interactions in the Nek9–Nek7 complex (Supplementary Fig. 1c). Interestingly, mutations Nek9^C808A^, Nek9^M824A^ and Nek9^P825A^ showed a reduction in Nek7 binding suggesting these residues contribute to the interaction but are not essential. Consistent with these data, independent experiments showed that a fragment of Nek9 comprising residues 750–859 retained binding to both Nek6 and Nek7, while further truncation at the C terminus (aa 750–826) showed reduced binding (Supplementary Fig. 3). Mutations in Nek9 W811, L816 and F821 in this fragment also disrupted the interaction with Nek6 and Nek7. Taken together, these findings confirm the identification of the binding site for Nek6 and Nek7 on Nek9.

### Crystal structure of a Nek7^Y97F^–Nek9^810–828^ complex

**Figure 1:**
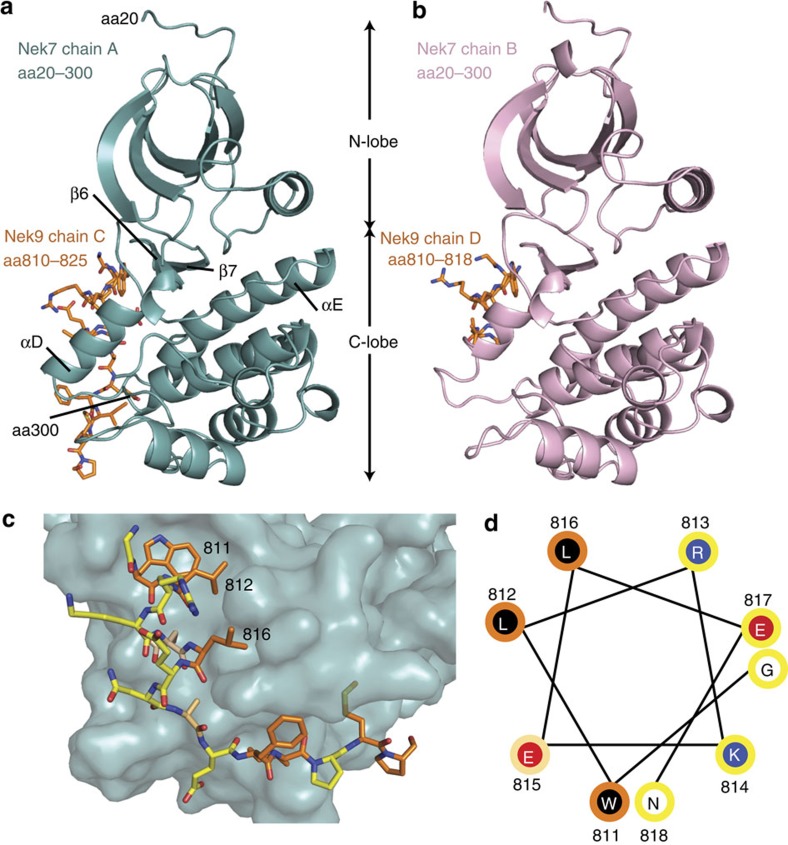
The crystal structure of Nek7 bound to Nek9. (**a**) Cartoon representation of Nek7 (teal, chain A) and stick representation of Nek9 810–825 (orange chain C). (**b**) Cartoon representation of Nek7 (pink, chain B) and Nek9 810–818 (orange, chain D). (**c**) Surface representation of Nek7 (teal, chain A) and stick representation of Nek9 810–825 (chain C). Carbon atoms in Nek9 are colour-coded according the results of the mutagenesis/co-precipitation data (Supplementary Fig. 1b,c): key binding residues (orange), non-essential residues (yellow) and untested residues (wheat). (**d**) Helical wheel plot of the Nek9 α-helix with residues colour-coded by chemical properties (inner circle: basic, blue; acidic, red; bulky hydrophobic, black; other, white) and contribution to Nek7 binding (outer circle, coloured as carbon atoms in **c**).

Having mapped the minimal binding region, we resolved to determine the structural basis of the interaction by protein crystallography using full-length Nek7 in the presence of an excess of synthetic peptide corresponding to aa 810–828 of Nek9. To obtain suitable crystals, we trialled several variants of Nek7, including WT and activating mutants of Nek7 (Y97A and Y97F). Crystals of Nek7^Y97F^–Nek9^810–828^ with a space group P3_1_ 2 1, which was different to the original Nek7^WT^ structure (space group I2 2 2, PDB code 2WQM), diffracted to 2.78 Å resolution. Molecular replacement using Nek7^WT^ as a search model revealed two chains of Nek7^Y97F^ in the asymmetric unit (Table 1, Fig. 1a,b). Residues 20–300 of Nek7^Y97F^ were modelled with the exception of the activation loop residues 182–195 (chain A) and 181–199 (chain B), which were disordered. The electron density maps in the vicinity of residue 97 in chain B clearly indicated a different conformation from the Nek7^WT^ structure (Supplementary Fig. 4a). Additional electron density was observed on the surface of Nek7^Y97F^ at the same position on both chains (Supplementary Fig. 4b–g). This was modelled as residues 810–825 (chain C) and 810–818 (chain D) of Nek9, bound to chains A and B of Nek7^Y97F^, respectively (Fig. 1a,b).

The first striking feature of this structure was that the binding site for Nek9 was not where we expected it to be. Based on the crystal structures of other kinase-activator complexes, such as Cyclin/CDK and Aurora-A/TPX2, we hypothesized that Nek9 would bind on the N-lobe close to the αC-helix and the β4-strand in which Tyr97 is located^31^. The crystal structure of the Nek7^Y97F^–Nek9^810–828^ complex revealed a binding site on the C-lobe of Nek7^Y97F^, within a groove formed by the hinge region, αD, αE and β6/β7. This site overlaps with an equivalent binding site for a docking motif that brings activating binding partners together with MAPK family members such as ERK2 and FUS3, although the overall regions of the kinase surfaces involved are distinct (Supplementary Fig. 5a,b)^32^,^33^. The site is occupied by a crystal contact in the crystal structure of Nek7 alone, which likely explains why the complex crystallizes in a different crystal form (Supplementary Fig. 5c). The site is close to the native C terminus of Nek7, and an equivalent pocket between αD, αE and β6/β7 is occupied by C-terminal extensions to the catalytic domain of many other kinases (Supplementary Fig. 5d)^34^,^35^,^36^,^37^.

**Figure 2:**
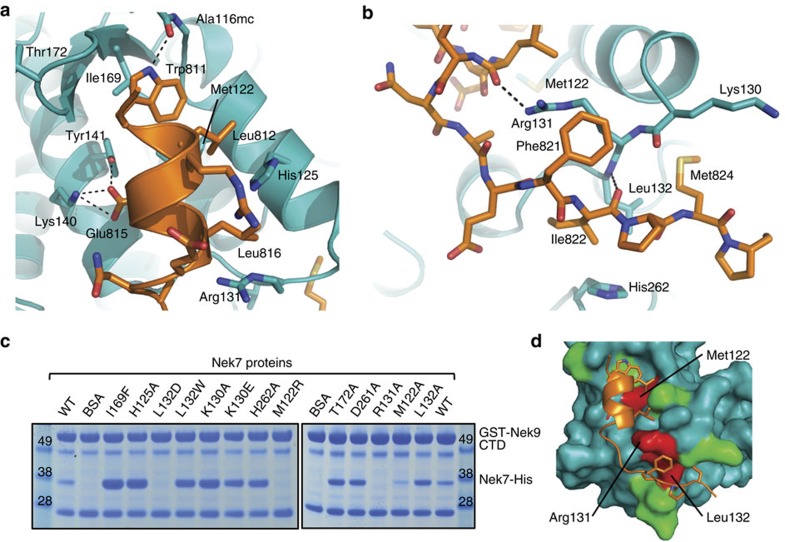
Detailed interactions within the Nek7–Nek9 interface. (**a**) Detailed view of the Nek7–Nek9 interaction in the region of Nek9 aa 810–816. (**b**) Detailed view of the Nek7–Nek9 interaction in the region of Nek9 aa 814–825. (**c**) Coomassie-stained SDS–PAGE gel showing the results of a GST co-precipitation experiment testing the binding of GST-Nek9-CTD to structure-based mutants of Nek7. (**d**) Mutagenesis/co-precipitation data from **c** mapped onto the surface of Nek7. Key binding residues (red), non-essential residues (green) and untested residues (teal).

In the structure of the complex, residues Nek9 810–818 form an α-helix (Fig. 1c,d). A set of hydrophobic residues (Trp811, Leu812 and Leu816) that were shown by co-precipitation assay to be crucial for the interaction are on one face of the helix and fit into the groove on the surface of Nek7, burying their side chains. The side chain of Nek9 Trp811 makes an additional H-bond with the carbonyl main chain of Ala116 within the hinge region of Nek7 (Fig. 2a). The other residue on this face is Glu815, which makes a substantial contribution to the interaction through formation of a salt bridge and a H-bond with the side chains of Lys140 and Tyr141, respectively, of Nek7. Glu815 was not tested in the alanine-scanning study because the Nek9^E815A^ protein was insoluble. The other face of the Nek9 α-helix has three charged residues, none of which contribute to the interface in the crystal structure or to binding by co-precipitation.

The C terminus of the Nek9 α-helix is capped by the side chain of Nek7 Arg131 (Fig. 2b). There is electron density corresponding to residues C terminal to the helix in both chains of Nek9, however we only felt confident in modelling this region in chain C. This region forms an extended structure that interacts with the linker between the αD and αE of Nek7. The side chains of Nek9 Ile822 and Met824 are substantially buried, one on either side of the side chain of Nek7 Leu132. One face of the phenyl ring of Phe821 packs onto the side chain of Nek7 Arg131. The only apparent polar interaction in this region is a putative H-bond between main-chain carbonyl of Nek9 Ile822 and main-chain amide of Nek7 Leu132. Thus, the side chain interactions of Nek9 present in the crystal structure are consistent with the results obtained from the binding assays.

To validate the crystal structure, we generated mutations in Nek7 to disrupt the interaction with Nek9. We identified amino acids on Nek7 that contact Nek9 and made mutations guided by the structure involving reversing charges, mutations to alanine or replacing hydrophobic amino acids. Generating mutations that disrupt the hydrophobic contact in the groove was more challenging because many of these residues are buried in the Nek7 structure and, for instance, Tyr141 mutations resulted in insoluble protein. GST co-precipitation binding assays were initially carried out using the minimal binding region of Nek9 (aa 810–828), and then replicated using the Nek9-CTD, with very similar results (Supplementary Fig. 6 and Fig. 2c). Of the mutations tested, R131A and L132D in the αD–αE linker appeared to completely abrogate the interaction, consistent with their extensive contacts with Nek9. Mutations of the αD-helix residue Met122 to either arginine or alanine also clearly reduced binding. Arg131, Leu132 and Met122 lie at the core of the interface and are absolutely conserved in human Nek6 (Fig. 2d and Supplementary Fig. 7). However, mutations we tested in residues at the periphery of the interface did not appear to disrupt the interaction (Fig. 2d). Thus, mutations in residues at the core of the interface in either Nek7 or Nek9 disrupt the interaction. We determined the kinase activity of phosphorylated, active forms of the Nek7 proteins with mutations at the core of the interface (Table 2 and Supplementary Fig. 8). Nek7^M122R^ and Nek7^L132D^ retained almost WT levels of activity as indicated by *k*_cat_/*K*_m_, and both Nek7^M122A^ and Nek7^R131A^ had almost 50% of WT activity. Hence, mutations in the core of the Nek9 binding interface do not interfere with Nek7 function and the loss of binding can be attributed to loss of specific interactions.

### Nek7 activation requires Nek9 self-association

Previous work has shown that addition of the Nek9-CTD increases kinase activity of initially unphosphorylated Nek7^WT^ but does not increase the activity of phosphorylated Nek7^WT^ or Nek7^Y97A^ (refs 27, 31, 38). Having identified the binding interaction, we carried out a series of *in vitro* kinase assays to investigate the activation mechanism of Nek7 by the Nek9-CTD. First, the synthetic peptide that was used for crystallography was incubated with initially unphosphorylated Nek7^WT^, Nek7^Y97A^ and Nek7^Y97F^ (Supplementary Fig. 9a). Nek7^Y97A^ and Nek7^Y97F^ have substantially increased activity towards substrate, as we have previously observed^31^, and these activated mutants also have higher autophosphorylation activity. However, the synthetic Nek9^810–828^ peptide had no effect on the activity of Nek7^WT^, Nek7^Y97A^ or Nek7^Y97F^. Hence, although residues 810–828 of Nek9 are necessary and sufficient for binding Nek7, further sequences within the Nek9-CTD are required for Nek7 activation.

**Figure 3:**
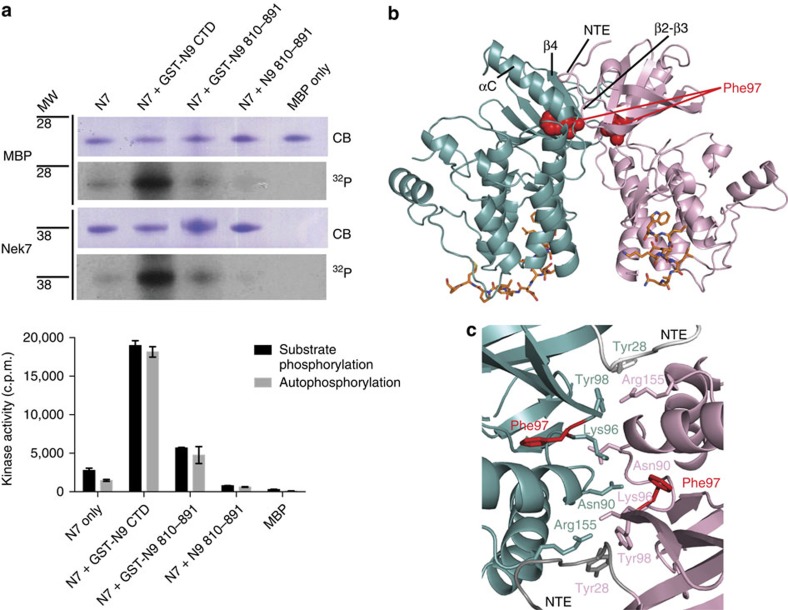
Nek9 self-association stimulates Nek7 activation. (**a**) *In vitro* kinase activity assay using a variety of Nek9 constructs to investigate the effect of Nek9 self-association on Nek7 autophosphorylation and substrate phosphorylation. The upper panel shows the Coomassie-stained gel (CB) and autoradiograph (^32^P). Incorporation of radioisotope was quantified by scintillation counting and is shown in the histogram (lower panel). Error bars represent the s.d. for two independent experiments. (**b**,**c**) Back-to-back dimerization of Nek7 is mediated through interactions between secondary structure elements, and the interface is centred around residue 97 (red).

We next explored the contribution of the CC region of the Nek9-CTD to Nek7 activation. A GST-tagged Nek9-CTD fragment lacking the CC (aa 810–891) was able to stimulate Nek7 activity, albeit not as efficiently as the full Nek9-CTD (Fig. 3a and Supplementary Fig. 9b). GST is itself a constitutive dimer, so we removed the GST tag to generate a monomeric fragment of the Nek9-CTD aa 810–891. This fragment did not stimulate Nek7 activity. We interpret these data to suggest that Nek9-CTD activation of Nek7 depends on self-association through the CC region, which can be mimicked by a dimeric GST tag.

We considered whether the activating Nek7 mutations at Tyr97 drive Nek7 self-association and thereby override the requirement for Nek9-CTD. GST co-precipitation binding assays showed that Nek7^Y97A^, Nek7^Y97F^ and phosphorylated Nek7^WT^ bind at least as well to Nek9-CTD as unphosphorylated Nek7^WT^ (Supplementary Fig. 10a,b). There was a slight increase in Nek9-CTD binding by phospho-Nek7^WT^ and the Nek7 Tyr97 mutants, which might result from increased dimerization of these proteins compared with non-phosphorylated Nek7. However, both Nek7^Y97A^ and Nek7^Y97F^ were purified as monomers by size-exclusion chromatography (Supplementary Fig. 10c). Therefore, we concluded that mutations in Tyr97 do not result in constitutive dimerization, nor do they override the ability to bind Nek9-CTD, leading us to investigate the structural basis of Nek7 activation by dimerization or mutations at Tyr97.

### Back-to-back dimerization is coupled to structural changes

On closer inspection of the Nek7^Y97F^–Nek9^810–828^ crystal structure, it was evident that the two Nek7 molecules present in the asymmetric unit formed a back-to-back dimer conformation centred around residue 97, in which the ATP binding pockets of the protomers face outwards, away from each other (Fig. 3b). The back-to-back interface is almost exclusively formed from interactions between the N-lobes, involving residues within the NTE (aa 24–29), β2-β3 loop (aa 55–58), αC-helix (aa 81, 84–88), αC-β4 loop (aa 89–92, 96) and β4-strand (aa 97, 98; Fig. 3c). There is a minor contribution from residues within the C-lobe, specifically in αE (154–156). These regions from both protomers participate in the interaction, but the precise molecular contacts these regions make differ between the two protomers, and the dimerization interface is asymmetric. The buried surface area of the interface is ∼780 Å^2^, slightly smaller than similar back-to-back interfaces (PERK 970 Å^2^ and PKR 900 Å^2^), consistent with a weak interaction^39^.

**Figure 4:**
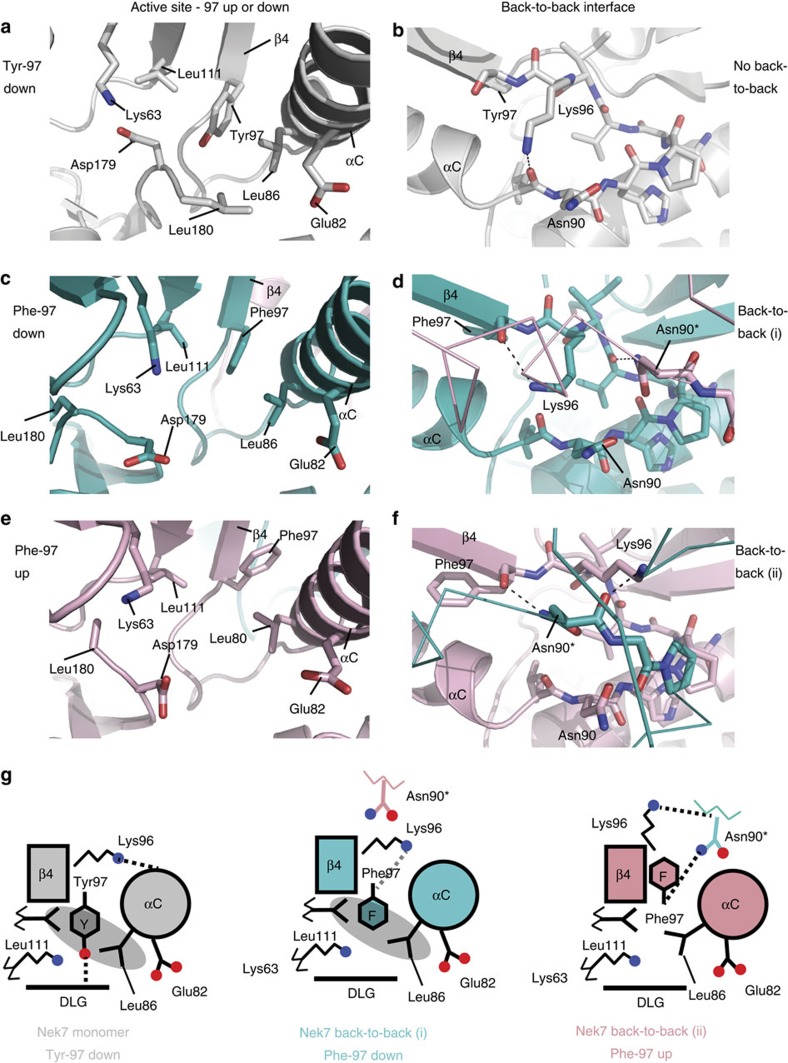
Relationship between the conformation of residue 97 and the back-to-back interface. (**a**,**c**,**e**) Active site viewed centred on residue 97. (**b**,**d**,**f**) View of the αC-β4 region that is involved in the back-to-back interface. In panels (**d**) and (**f**), the dimeric partner is shown as a thin ribbon with key residues shown as in sticks. The asterisk on Asn90* indicates that it belongs to the dimeric partner. Structures shown are: (**a**,**d**) Nek7^WT^ alone (PDB code 2WQN, coloured grey); (**b**,**e**) Nek7^Y97F^, chain A (coloured teal); (**c**,**f**) Nek7 ^Y97F^, chain B (coloured pink). (**g**) Schematic illustrations of the coupling between the conformation of residue 97 and the back-to-back interface.

We compared the structures of Nek7^WT^ and the two different chains of Nek7^Y97F^ in the complex with Nek9^810–828^ (Fig. 4). In Nek7^WT^, the kinase is locked in an inactive conformation (‘Tyr-down’), characterized by a disordered activation loop, outward position of the αC-helix, inactive positions of R-spine residues, Tyr97 (β4-strand), Leu86 (αC-helix), Leu180 (DLG motif) and His159 (HRD motif), and the absence of a salt bridge between Lys63 and Glu82 (Fig. 4a). We hypothesized that this conformation is stabilized by the position of Tyr97 in two ways: a H-bond between the hydroxyl group of Tyr97 and the backbone amide of Leu180; and hydrophobic contacts involving the aromatic ring of Tyr97 that is sandwiched between Leu86 located on the αC-helix and the gatekeeper residue Leu111. There is no back-to-back interface in the original Nek7 structure, and the main chain of Tyr97 is partially occluded by the side chain of Lys96 (Fig. 4b).

Chain A of Nek7^Y97F^ bound to Nek9^810–828^ closely resembles the original Nek7 structure (Fig. 4c). Phe97 is in the ‘down’ position, and thus the αC-helix is stabilized in a similar position to that observed in the original Nek7 structure. However, the H-bond between this side chain and the main chain of the activation loop cannot be formed and the two regions have moved apart. The side chain of Phe97 has moved ∼2 Å further from the active site and the activation loop is in a different orientation with flipped positions of Leu180 and Asp179. Most of the activation loop is disordered, as in the original Nek7 structure. The conformation of residues in the back-to-back interface is also very similar to the Nek7^WT^ structure (Fig. 4d).

In contrast, there are several notable conformational changes in chain B of Nek7^Y97F^ bound to Nek9^810–828^ (Fig. 4e). Most prominently, Phe97 is in the ‘up’ position. The αC-helix has moved, bringing Leu86 closer to the gatekeeper residue Leu111 and Glu82 closer to its salt bridge partner Lys63. Thus, these key residues are shifted towards the positions that are expected in an active kinase. The back-to-back interface of chain B shows a clear change in the position of Lys96, which undergoes main- and side-chain motions to accommodate Asn90 from chain A close to the main chain of residue 97 (Fig. 4f).

Mutations at Tyr97 stimulate the activity of initially unphosphorylated Nek7, but do not increase the catalytic activity of active, phosphorylated Nek7 (Table 2 and Supplementary Fig. 9a). We take this to indicate that these mutations stimulate Nek7 activity through release of autoinhibition, which increases the basal activity of the kinase to promote autophosphorylation. The structure of Nek7^Y97F^ in two different conformations show how autoinhibition is released by this mutation in a two step process (Fig. 4g). First, the loss of a H- bond weakens the association of the residue 97 side chain with the activation loop. Second, the side chain of residue 97 is flipped into the ‘up’ position through a rotamer change and through conformational changes in the main chain at residues 96 and 97. Comparing the conformations of chains A and B, the way in which Asn90 interacts with the αC-β4 linker of the other protomer is coupled to the position of residue 97. The conformational differences in this region between chains A and B comprise both main-chain and side-chain motions, which are necessary to accommodate the side chain of Asn90. Thus, the different ways in which Asn90 of chains A and B interact with the αC-β4 linker of the partner protomer are coupled to the different orientations of residue 97 in those chains. This suggested to us that back-to-back dimerization of Nek7 might be coupled to induction of the ‘up’ position of residue 97, providing an explanation as to how dimerization of Nek9 could induce Nek7 activation by promoting correctly oriented self-association.

### Mutations in the back-to-back interface reduce activity

**Figure 5:**
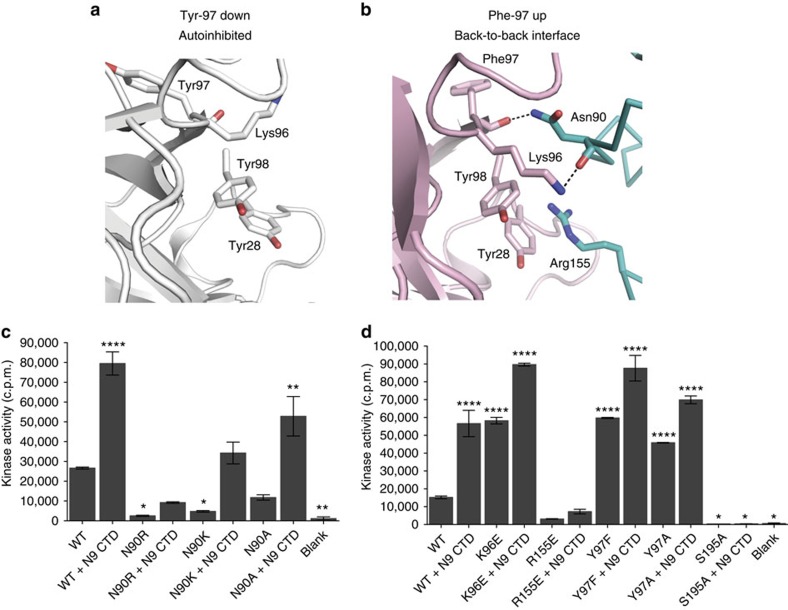
Mutations in the Nek7 back-to-back interface. (**a**) Structure of autoinhibited Nek7 showing residues involved in the back-to-back interface. (**b**) Structure of Nek7 in the back-to-back conformation. (**c**,**d**) *In vitro* kinase activity assay of WT Nek7 and Nek7 mutants alone and in the presence of Nek9-CTD. Kinase activity was quantified by scintillation counting. Error bars represent the standard error for two independent reactions. *P<0.05, **P<0.01 and ****P<0.0001 using one-way analysis of variance with Dunnett’s *post hoc* test compared with the WT reaction for Nek7 alone reactions or to the WT plus N9 CTD reaction for assays performed in the presence of Nek9-CTD.

To investigate the role of back-to-back dimerization in Nek7 activation we generated point mutations in the interface and tested the activity of the initially unphosphorylated protein in the presence or absence of Nek9-CTD (Fig. 5). All mutants retained binding to Nek9-CTD and exhibited clear melting curves, showing that the mutations did not affect the gross folding of Nek7 (Supplementary Fig. 11a,b,c).

Mutation of Asn90 to arginine or lysine strongly reduced activity in the presence or absence of Nek9-CTD (up to tenfold), while mutation of Asn90 to alanine reduced activity by a factor of ∼2 (Fig. 5c). These results are consistent with the structural model because Asn90 is buried in the back-to-back interface, and so mutation to a bulky residue might be expected to disrupt the interface, whereas mutation to a smaller residue such as alanine might be expected to have a lesser impact. In contrast, mutation of Lys96 to glutamic acid was strongly activating, similar to mutations at Tyr97 (Fig. 5d). This mutation would disrupt interactions with main-chain carbonyls that maintain Lys96 in a position that occludes the main chain of Tyr97 in autoinhibited conformations of Nek7.

There is no salt bridge in the Nek7 back-to-back interface, unlike those found in kinases such as PERK and IRE1 (Supplementary Fig. 12). Instead, we identified an interaction between Arg155 and an aromatic cage consisting of the side chains of Tyr28 in the NTE and Tyr98 (Fig. 5b). Disruption of this interaction by mutation of Arg155 strongly reduced Nek7 activation (Fig. 5d). We have previously found that deletion of the NTE of Nek7, specifically residues 20–30, or a double point mutant (Tyr28Ala and Leu31Ala), reduces kinase activity^31^. Taken together, these data support our model that the back-to-back interface contributes to kinase activation^31^.

**Figure 6:**
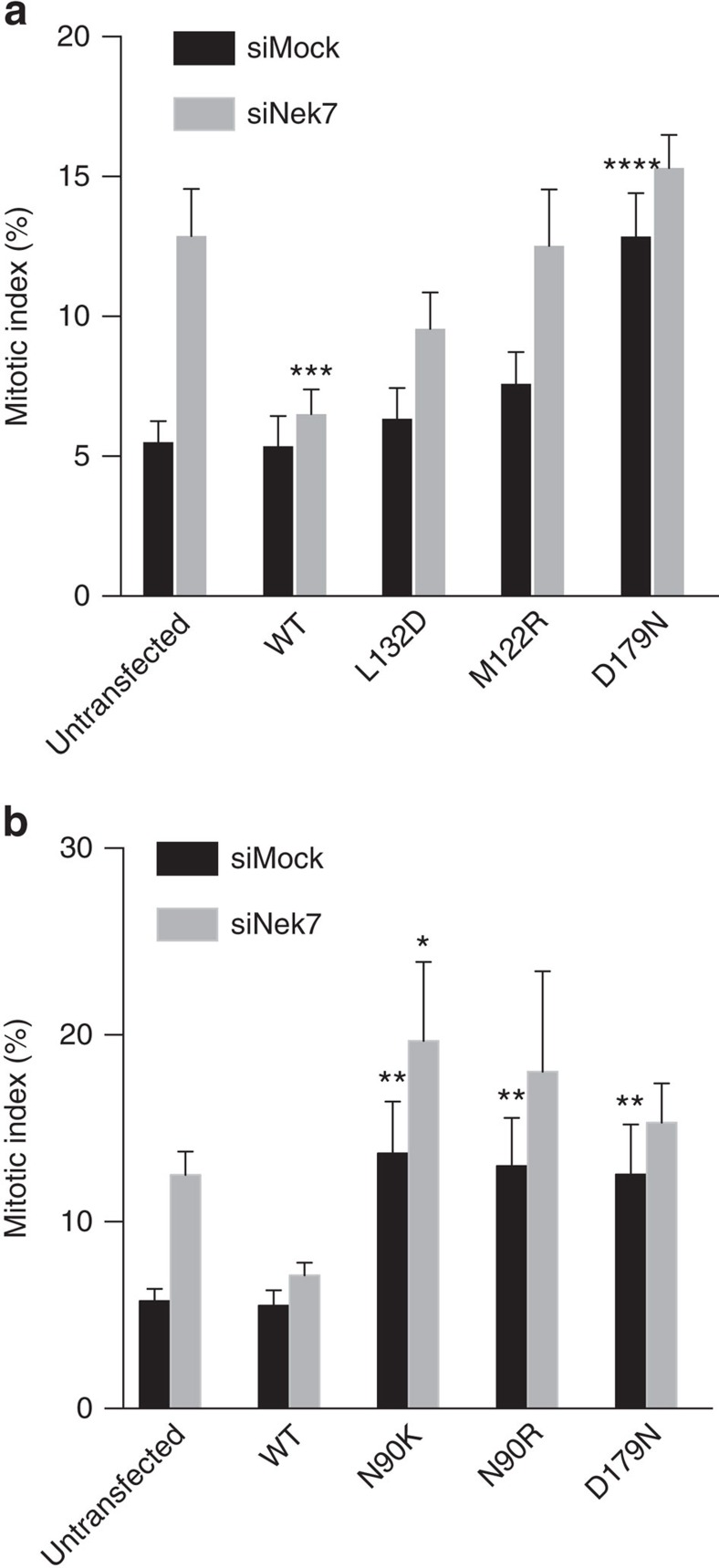
Biological relevance of Nek7 allosteric activation by Nek9. (**a**,**b**) HeLa cells were transfected with RNAi resistant Flag-Nek7 WT or binding-deficient mutants as indicated, and grown in selection media containing G418 for 7 days to enrich the transfected cell population. Cells were then either mock- or Nek7-depleted for 72 h before being fixed and processed for immunofluorescence microscopy with Flag and phospho-H3 antibodies. The mitotic index of transfected cells was counted. Data represent mean (±s.d.) for three separate experiments where *n*=100–200 cells. Statistical analyses were carried out using a one-way analysis of variance followed by *post hoc* testing. *P<0.05, **P<0.01 and ***P<0.005.

We investigated the biological relevance of the two protein–protein interactions using stably transfected siRNA-resistant Flag-tagged Nek7 WT or mutants in HeLa cells (Supplementary Fig. 11d). HeLa cells were mock- or siNek7-depleted and the mitotic index scored using phospho-histone H3 as a mitotic marker in fixed cells. In control cells, not transfected with a recombinant Nek7 plasmid, siNek7 treatment increased the mitotic index in line with our previous observations (Fig. 6a)^21^. Expression of Nek7^WT^ rescued the mitotic index, whereas expression of mutants defective in Nek9 binding, Nek7^L132D^ and Nek7^M122R^ did not significantly rescue the mitotic index (Fig. 6a). These two mutants slightly increased mitotic index in mock-depleted cells, but this effect was not significant. In contrast, Nek7^D179N^ a kinase-dead mutant has a dominant effect—the mitotic index is increased even in mock-depleted cells. Similar dominant effects were obtained for Nek7^N90R^ and Nek7^N90K^ mutants (Fig. 6b). Overall, these results suggest that mutations in the Nek9 binding site render Nek7 unable to function, but that there is no interference with the activity of endogenous Nek7. In contrast, Nek7 mutants that are kinase-inactive through mutation of the active site (D179N) or back-to-back dimer interface required for efficient activation (N90K and N90R), are not only non-functional, but also block the activity of endogenous Nek7 (and Nek6), presumably by sequestering Nek9.

## Discussion

We previously hypothesized that Nek9-CTD binding to Nek7 relieves its autoinhibition and allows Nek7 to form an active kinase^31^. On the basis that the interaction might alter the conformation of Tyr97, we speculated that the interaction would involve the αC-helix/β4-strand on the N-lobe of Nek7. In this study we elucidated the structural basis of the Nek7–Nek9 interaction, which occurs at an unexpected site on the C-lobe of Nek7, and discovered that dimeric Nek9 stimulates Nek7 autophosphorylation. We did not observe a fully active conformation of Nek7^Y97F^ and the presence of the activating mutation and the minimal Nek9 peptide are insufficient to induce this conformation, which most likely requires the phosphorylation of Ser195 in the activation loop. However, inspection of the active sites shows changes with respect to the original Nek7 structure consistent with progress along the pathway from autoinhibition towards an active state. Most prominently, chain B of Nek7^Y97F^ has residue 97 in the ‘up’ conformation, which is necessary for formation of an inward and active position of the αC-helix. The orientation of residue 97 is coupled to specific interactions at the back-to-back dimer interface.

Nek9 forms homo-oligomers through its CC domain^40^. We show that the CC is not required for binding to Nek7, but is clearly important in the activation of Nek7. The self-association properties of the CC domain are key to Nek7 activation because fusion of Nek9 to GST is able to substitute for the CC. However, Nek9-CTD with a CC is more efficient, suggesting that the precise structural arrangement it confers contributes to the optimal activation of Nek7. Indeed, Nek7^Y97A^, Nek7^Y97F^ and Nek7^K96E^ were more active in the presence of Nek9-CTD, suggesting that dimerization induced by Nek9-CTD can increase the efficiency of Nek7 activation, even when autoinhibition is relieved by mutation (Fig. 5d).

The structure of the Nek7^Y97F^–Nek9^810–828^ complex has a back-to-back dimerization interface that was not observed in the original Nek7^WT^ structure^31^. Back-to-back dimerization is unlikely to be directly induced by the Nek9^810–828^ peptide because the peptide does not stimulate activity and there is no obvious structural connection between the Nek9 binding site on Nek7 and the back-to-back interface. There is an indirect link because the Nek9^810–828^ peptide blocks a crystal packing site that was observed in the original crystal form of Nek7^WT^, and thus the crystals of the complex generate a new crystal form through alternative crystal packing arrangements, including the back-to-back dimer interface.

**Figure 7:**
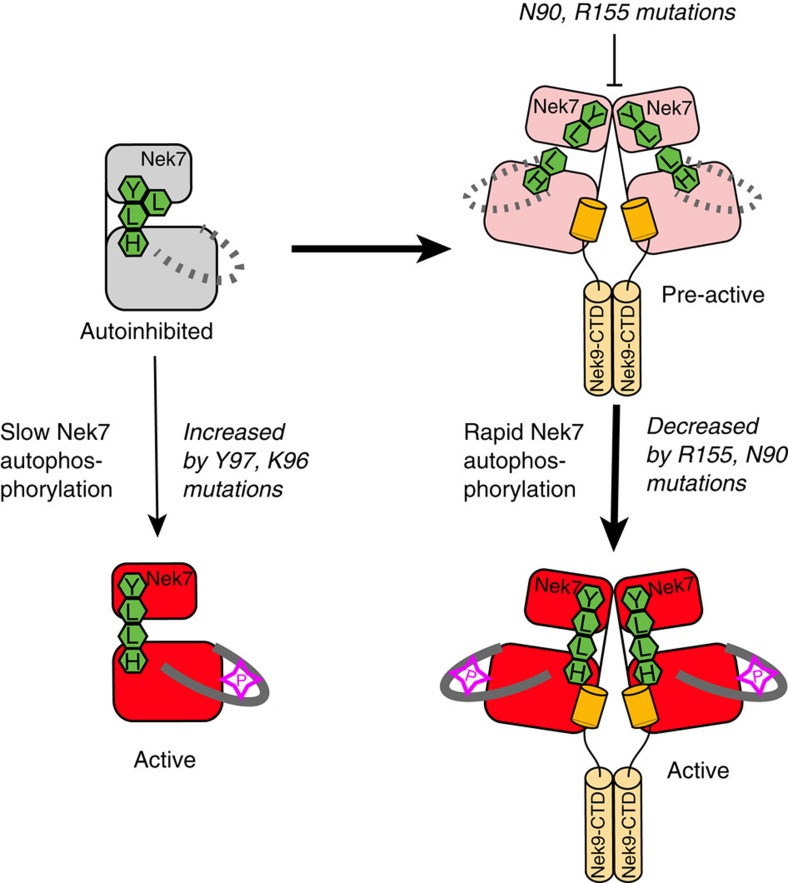
Schematic model of Nek7 activation by Nek9 binding and induced dimerization. The autoinhibited state of Nek7 (grey) is characterized by the Tyr-down conformation, in which the active site is blocked by a collapsed set of four R-spine residues (green). Autophosphorylation of Nek7 is slow and can be accelerated by mutation (Tyr97 and Lys96) or Nek9. Binding of self-associated Nek9 induces back-to-back dimerization of Nek7, which releases autoinhibition by promoting a Tyr-up conformation. Nek7 autophosphorylation results in an active kinase conformation. Once phosphorylated, Nek7 can remain bound to Nek9, but Nek9 is no longer required for Nek7 activity. Phosphorylation of Nek7 by Nek9 kinase activity is an alternative mechanism *in vivo.*

We have previously shown that the autoactivation of Nek7 alone is concentration-dependent, and therefore involves self-association, and that Nek9-CTD enhances activation^31^,^38^. Here we propose a mechanism to bring these observations together in the light of our new data: Nek9 self-association brings Nek7 protomers together as a back-to-back dimer that releases Nek7 autoinhibition through a set of specific interactions that switches the conformation of Tyr97. A scheme of this speculative mechanism is illustrated in Fig. 7. In this scheme, Nek9 induces a pre-active state in Nek7 characterized by the ability of the kinase to autophosphorylate efficiently. Autophosphorylation most likely occurs in trans between, not within, Nek7 back-to-back dimers. The model is consistent with the observed activation of Nek7 Tyr97 mutants that, in effect, already exist in this pre-active state because the block to autophosphorylation has been removed. The pre-active state resembles chain B of the Nek7^Y97F^–Nek9^810–828^ complex. However, there are outstanding questions to be resolved, such as whether the activating back-to-back interface is asymmetric as observed in the structure of Nek7^Y97F^–Nek9^810–828^. It also remains to be determined whether the primary source of activation loop phosphorylation of Nek6 and Nek7 *in vivo* is a result of autophosphorylation or phosphorylation by Nek9.

Activation through dimerization is a common theme in the regulation of protein kinases. The importance of back-to-back dimerization in a number of different protein kinases has recently been highlighted and there is a clear link with distorted R-spine/αC-helix in kinase-inactive forms^4^. PKR and PERK, two members of the eIF2α kinase family, crystallize in a back-to-back conformation that, like the Nek7 dimer, is almost entirely formed through interactions between their N-lobes (Supplementary Fig. 12d). Structure–function studies verified that back-to-back dimerization is crucial for activation of PKR and PERK^13^,^15^. Notably, both of these kinases have a Tyr residue at the equivalent position of Nek7 Tyr97. In the PKR and PERK dimeric structures, the side chain forms a H-bond with the side chain of an Asp residue from the opposing chain of the dimer. Inactive structures are not available for PKR and PERK, but it has been suggested that they would reveal a distortion of the αC-helix and R-spine based on structures of other kinases in this family. In contrast, an alternative back-to-back dimer arrangement has been observed in the eIF2α kinase GCN2, in which one protomer is rotated by ∼180**°** (Supplementary Fig. 12e). Tyr658, which is the equivalent of Tyr97 of Nek7, points into the active site and forms a H-bond with the main chain of the Phe854 in the DFG motif. Thus, GCN2 adopts a Tyr-down conformation similar to Nek7, as part of an inactive conformation in which the R-spine is disrupted and the Lys–Glu salt bridge are broken.

A further example of regulation through back-to-back dimerization is found in the kinase-endoribonuclease IRE1. Crystal structures of active IRE1 from *Saccharomyces cerevisiae* and pre-active human IRE1 in apo form reveal a back-to-back dimer that primarily involves the kinase N-lobe, with further contributions from an extended loop between strands β6 and β7 (Supplementary Fig. 12c)^16^,^17^,^41^. IRE1 has a Tyr residue at the equivalent position of Nek7 Tyr97 (Tyr628 in human IRE1) and, in the back-to-back dimer structure, there are extensive interactions between the protomers in this region. The structure of human IRE1 bound to ADP reveals a different arrangement, a face-to-face dimer that does not involve residues in the vicinity of Tyr628. In this form, the kinase domain of IRE1 resembles an inactive kinase with distortions to the R-spine and αC-helix and Tyr628 points into the active site and forms a H-bond with the activation loop at the DFG motif^4^,^41^. Mutation of Tyr628 enhances IRE1 autophosphorylation, which is equivalent to the findings in Nek7 (ref. 41). We consider the conservation of a Tyr residue in the β4-strand among kinases that form back-to-back dimers to be remarkable. These other kinases with back-to-back dimer interfaces are membrane-associated and have additional domains that drive self-association. What distinguishes Nek7 from these other kinases is that it is cytosolic and does not have a domain that drives its self-association, but relies on induced dimerization on interaction with Nek9.

While Nek6 and Nek7 require interaction with Nek9 to induce its dimerization, six other human Neks contain CC regions that are likely to confer homo-oligomerization and in Nek2 a leucine-zipper domain is crucial for kinase activity^20^,^42^. Activation through dimerization is likely to be an important feature of regulation for Nek kinases. However, the back-to-back interface residues are conserved in only a subset of other human Neks (Supplementary Fig. 13). Therefore, further studies will be required to clarify the mechanisms by which other Neks are regulated through dimerization.

## Methods

### Protein expression and purification

The expression constructs for full-length Nek7 (in pET30, C-terminal His_6_-tag) and Nek9-CTD (in pGEX-4T1, N-terminal GST tag) have been previously described^31^. Nek7 proteins were co-expressed with or without λ-phosphatase (in pCDF-Duet, untagged^43^) overnight in Codonplus RPIL (Stratagene) *Escherichia coli* at 18 °C following induction with 0.6 mM isopropyl-beta-D-thiogalactopyranoside. Cells were lysed by sonication, clarified, and Nek7 protein was initially purified using a 5 ml HisTRAP column (GE Healthcare) equilibrated in 50 mM HEPES (pH 7.5), 300 mM NaCl, 5% glycerol and a 20–250 mM imidazole gradient. The final gel filtration step of Nek7 purification was performed using a Superdex-200 16/60 column which was equilibrated in 50 mM HEPES pH 7.5, 300 mM NaCl, 5% glycerol and 5 mM dithiothreitol. Nek9 protein expression in *E. coli* was induced by addition of 0.6 mM isopropyl-beta-D-thiogalactopyranoside followed by incubation overnight at 18 **°**C, and proteins were purified using glutathione sepharose chromatography according to the manufacturer’s instructions (GE Healthcare). Nek9-CTD WT and mutants were dialysed after elution from glutathione sepharose beads in 50 mM HEPES pH 7.5, 300 mM NaCl, 5% glycerol and 5 mM β-mercaptoethanol.

For kinase assays where total incorporation of radioisotope was measured, the GST-tag on Nek9-CTD was removed by overnight cleavage with 3C protease. The protein was subjected to Q-Sepharose chromatography as per the manufacturer’s instructions (GE Healthcare) followed by size-exclusion chromatography into Nek7 gel filtration buffer.

Site directed mutagenesis for both Nek7 and Nek9 was performed according to the Quikchange protocol (Stratagene) and mutations were confirmed by DNA sequencing (MWG Eurofins).

### Protein crystallization

Nek7^Y97F^ was co-crystallized with a synthetic peptide of Nek9^810–828^ (Peptide Synthetics, dissolved in Nek7 gel filtration buffer) by sitting drop vapour diffusion. Drops were composed of Nek7 protein at 440 μM and Nek9 peptide at 880 μM, mixed 1:1 with the reservoir buffer (0.2 M potassium thiocyanate, 0.1M bis-Tris propane pH 7.5, 20% (w/v) PEG 3350). Crystals were briefly soaked in cryoprotectant solution containing 20–25% ethylene glycol and flash frozen in liquid nitrogen.

### Crystallography

Diffraction data was collected at Diamond Light Source, Didcot, UK at beamline I03 at a temperature of 100 K and a wavelength of 0.979 Å. Auto processed data were used directly for molecular replacement using PHASER^44^ with the original Nek7-APO structure (PDB 2WQM (ref. 31)) as the model and the N- and C-lobes were independently searched for. A total of four protein chains in the asymmetric unit were identified: two Nek7^Y97F^ and two Nek9^810–828^. Subsequent iterative refinement cycles were performed using PHENIX^45^ and COOT^46^, and final cycles of refinement were carried out using BUSTER^47^,^48^. Geometry was good (95% Ramachandran favoured, 0% outliers) as assessed using MolProbity^49^. Buried surface areas were calculated using PDBePISA^50^.

### *In vitro* kinase assays

Nek7 and Nek9 were pre-incubated in kinase buffer (50 mM HEPES.KOH pH 7.4, 5 mM MnCl_2_, 5 mM β-glycerophosphate, 5 mM NaF and 1 mM dithiothreitol) and with the substrate myelin basic protein, for 1 h at room temperature. Following pre-incubation, 4 μM unlabelled ATP and 1 mCi radiolabelled ^32^P-ATP were added to the reaction mixture. Kinase reactions were carried out for 30 min at 30 °C with shaking and terminated by the addition of SDS-loading buffer. Samples were subsequently analysed by SDS–PAGE and autoradiography. Incorporation of ^32^P into substrate and Nek7 (by autophosphorylation) was determined by scintillation counting.

For the analysis of back-to-back dimer mutations, total incorporation of radioisotope was measured by scintillation counting. Kinase reactions were set-up as stated above and incubated at room temperature for 45 min. Reactions were terminated by the addition of 2% orthophosphoric acid. Samples were pipetted onto P81 paper and allowed to air-dry. Unincorporated ^32^P-ATP was removed by extensive washing with 0.2% orthophosphoric acid. Statistical analysis was carried in Prism (Graphpad Software, Inc.). One-way analysis of variance was performed to identify significant changes followed by Dunnett’s *post hoc* analysis.

### GST co-precipitation assays

100 μg of GST-Nek9 mutant proteins and GST (control) were immobilized onto 30 μl of glutathione sepharose-4B beads (GE Healthcare) equilibrated in 50 mM HEPES pH 7.5, 300 mM NaCl, 5% glycerol, 5 mM dithiothreitol and 0.05% Tween-20 for 1 h at 4 °C. Immobilized beads were washed three times with 500 μl buffer and subsequently incubated with 100 μg Nek7 in 500 μl buffer for 1 h at 4 °C. After three final washes with buffer, the resin was resuspended in 30 μl buffer and 30 μl SDS-loading buffer. Samples were resolved by SDS–PAGE and visualized by either Coomassie staining or by immunoblotting using an α-His_6_ antibody (Clontech).

### Determination of active Nek7 kinetic parameters

Nek7 mobility shift assays were performed at room temperature in 100 mM HEPES (pH 7.5), 0.003% Brij-35, 0.004% Tween-20 and 10 mM MgCl_2_ with 3 μM-labelled peptide (5-FAM-FLAKSFGSPNRAYKK, Caliper peptide 32). ATP dilutions were obtained by serial dilution and reactions started by the addition of enzyme solution to a premix of the other reagents. Reactions were followed in duplicate in 30 μl volumes with a Caliper EZ Reader II system (Perkin Elmer). For the calculation of *k*_cat_ and *K*_m_, initial rates were determined from the kinetic data and plotted against ATP concentration; the resulting curves were fitted to the equation *Y*=*Et* × *k*_cat_ × *X*/(*K*_m_+*X*) using GraphPad Prism software, where *X* is the ATP concentration, *Y* is enzyme velocity and *Et* is the concentration of Nek7.

### Cell culture and microscopy

HeLa cells, obtained from the ATCC, were grown in Dulbecco’s modified Eagle’s medium (Invitrogen) supplemented with 10% heat-inactivated fetal bovine serum (FBS), 100 IU ml^−1^ penicillin and 100 μg ml^−1^ streptomycin at 37 °C in a 5% CO_2_ atmosphere. Transient transfections were performed with Fugene HD reagent (Fugene) according to manufacturer’s instructions and cells were transfected for 24 h, after which they were maintained for 7 days in Dulbecco’s modified Eagle’s medium supplemented with 800 μg ml^−1^ G418. For siRNA depletion, cells at 30–40% confluency were cultured in opti-MEM reduced serum medium and transfected with 50 nM Nek7 ON-TARGETplus siRNA duplexes (O’Regan and Fry, 2009) using Oligofectamine (Invitrogen, UK) according to manufacturer’s instructions. Seventy-two hours after transfection, cells were fixed for immunocytochemistry. Briefly, following aspiration of media and cells were fixed by incubation in ice-cold methanol at −20 °C for 30 min. Cells were then blocked for 30 min in PBS supplemented with 3% bovine serum albumin before being incubated with the required antibody diluted as appropriate in blocking solution. Primary antibodies were mouse Flag (0.5 μg ml^−1^; Sigma, Cat. # F1804) and rabbit phospho-histone H3 (2 μg ml^−1^; MerckMillipore, Cat. # 06-570). Secondary antibodies used were Alexa Fluor 488 and 594 goat anti-rabbit and goat anti-mouse IgGs (1 μg ml^−1^; Invitrogen, Cat. # A11008 and A11005, respectively).

## Additional information

**Accession codes:** Coordinates and reflection data files have been submitted to the protein data bank with accession number 5DE2.

**How to cite this article:** Haq, T. *et al.* Mechanistic basis of Nek7 activation through Nek9 binding and induced dimerization. *Nat. Commun.* 6:8771 doi: 10.1038/ncomms9771 (2015).

## Supplementary Material

Supplementary Information Supplementary Figures 1-13

## Figures and Tables

**Table 1:** Data collection and refinement statistics.

*Data collection*	
Space group	P3_1_ 2 1
Cell dimensions	
*a*, *b*, *c* (Å)	88.12, 88.12, 155.61
α, β, γ (**°**)	90.00, 90.00, 120.00
Resolution (Å)	54.48–2.78 (2.85–2.78)*
*R*_merge_	0.108 (0.964)
R_pim_	0.037 (0.329)
I/σI	17.3 (2.6)
CC_1/2_	0.999 (0.809)
Completeness (%)	99.9 (99.8)
Redundancy	9.5 (9.5)
	
*Refinement*	
Resolution (Å)	54.48–2.78
No. reflections	18191
R_work_/R_free_	0.206/0.254
No. atoms	
Protein	4442
Water	10
*B*-factor	
Wilson	82.8
Protein	66.4
Water	51.7
R.m.s. deviations	
Bond lengths (Å)	0.009
Bond angles (°)	1.02

^*^Highest resolution shell is shown in parenthesis.

**Table 2:** Summary of active Nek7 kinetic parameters.

**Nek7**	***k***_**cat**_ **(**_**min**_^−**1**^**)**	**ATP** ***K***_**m**_ **(mM)**	***k***_**cat**_ **/** ***K***_**m**_	**Rel** ***k***_**cat**_**/*****K***_**m**_
WT	1.212±0.011	0.00787±0.00038	153.924	1.000
M122A	0.452±0.0054	0.00666±0.00044	67.959	0.442
M122R	0.697±0.0072	0.0052±0.00031	134.064	0.871
R131A	0.669±0.0042	0.00922±0.00032	72.578	0.472
L132D	0.8601±0.026	0.00562±0.00097	153.076	0.994
Y97A	0.194±0.0034	0.00923±0.00088	20.967	0.136
Y97F	0.36±0.0039	0.0109±0.00063	33.119	0.215

## References

[b1] Jura N. *et al.* Catalytic control in the EGF receptor and its connection to general kinase regulatory mechanisms. Mol. Cell 42, 9–22 (2011). 21474065 10.1016/j.molcel.2011.03.004 PMC3175429

[b2] Endicott J. A., Noble M. E. & Johnson L. N. The structural basis for control of eukaryotic protein kinases. Annu. Rev. Biochem. 81, 587–613 (2012). 22482904 10.1146/annurev-biochem-052410-090317

[b3] Bayliss R., Fry A., Haq T. & Yeoh S. On the molecular mechanisms of mitotic kinase activation. Open Biol. 2, 120136 (2012). 23226601 10.1098/rsob.120136 PMC3513839

[b4] Lavoie H., Li J. J., Thevakumaran N., Therrien M. & Sicheri F. Dimerization-induced allostery in protein kinase regulation. Trends Biochem. Sci. 39, 475–486 (2014). 25220378 10.1016/j.tibs.2014.08.004

[b5] Johnson L. N. & Lewis R. J. Structural basis for control by phosphorylation. Chem. Rev. 101, 2209–2242 (2001). 11749371 10.1021/cr000225s

[b6] Nolen B., Taylor S. & Ghosh G. Regulation of protein kinases; controlling activity through activation segment conformation. Mol. Cell 15, 661–675 (2004). 15350212 10.1016/j.molcel.2004.08.024

[b7] De Bondt H. L. *et al.* Crystal structure of cyclin-dependent kinase 2. Nature 363, 595–602 (1993). 8510751 10.1038/363595a0

[b8] Yamaguchi H. & Hendrickson W. A. Structural basis for activation of human lymphocyte kinase Lck upon tyrosine phosphorylation. Nature 384, 484–489 (1996). 8945479 10.1038/384484a0

[b9] Sicheri F., Moarefi I. & Kuriyan J. Crystal structure of the Src family tyrosine kinase Hck. Nature 385, 602–609 (1997). 9024658 10.1038/385602a0

[b10] Xu W., Doshi A., Lei M., Eck M. J. & Harrison S. C. Crystal structures of c-Src reveal features of its autoinhibitory mechanism. Mol. Cell 3, 629–638 (1999). 10360179 10.1016/s1097-2765(00)80356-1

[b11] Kornev A. P., Haste N. M., Taylor S. S. & Eyck L. F. Surface comparison of active and inactive protein kinases identifies a conserved activation mechanism. Proc. Natl Acad. Sci. USA 103, 17783–17788 (2006). 17095602 10.1073/pnas.0607656103 PMC1693824

[b12] Kornev A. P. & Taylor S. S. Defining the conserved internal architecture of a protein kinase. Biochim. Biophys. Acta 1804, 440–444 (2010). 19879387 10.1016/j.bbapap.2009.10.017 PMC3435107

[b13] Cui W., Li J., Ron D. & Sha B. The structure of the PERK kinase domain suggests the mechanism for its activation. Acta Crystallogr. D Biol. Crystallogr. 67, 423–428 (2011). 21543844 10.1107/S0907444911006445 PMC3087621

[b14] Dar A. C., Dever T. E. & Sicheri F. Higher-order substrate recognition of eIF2alpha by the RNA-dependent protein kinase PKR. Cell 122, 887–900 (2005). 16179258 10.1016/j.cell.2005.06.044

[b15] Dey M. *et al.* Mechanistic link between PKR dimerization, autophosphorylation, and eIF2alpha substrate recognition. Cell 122, 901–913 (2005). 16179259 10.1016/j.cell.2005.06.041

[b16] Korennykh A. V. *et al.* The unfolded protein response signals through high-order assembly of Ire1. Nature 457, 687–693 (2009). 19079236 10.1038/nature07661 PMC2846394

[b17] Lee K. P. *et al.* Structure of the dual enzyme Ire1 reveals the basis for catalysis and regulation in nonconventional RNA splicing. Cell 132, 89–100 (2008). 18191223 10.1016/j.cell.2007.10.057 PMC2276645

[b18] Padyana A. K., Qiu H., Roll-Mecak A., Hinnebusch A. G. & Burley S. K. Structural basis for autoinhibition and mutational activation of eukaryotic initiation factor 2alpha protein kinase GCN2. J. Biol. Chem. 280, 29289–29299 (2005). 15964839 10.1074/jbc.M504096200

[b19] Ali M. M. *et al.* Structure of the Ire1 autophosphorylation complex and implications for the unfolded protein response. EMBO J. 30, 894–905 (2011). 21317875 10.1038/emboj.2011.18 PMC3049214

[b20] Fry A. M., O'Regan L., Sabir S. R. & Bayliss R. Cell cycle regulation by the NEK family of protein kinases. J. Cell Sci. 125, 4423–4433 (2012). 23132929 10.1242/jcs.111195 PMC3500863

[b21] O'Regan L. & Fry A. M. The Nek6 and Nek7 protein kinases are required for robust mitotic spindle formation and cytokinesis. Mol. Cell. Biol. 29, 3975–3990 (2009). 19414596 10.1128/MCB.01867-08 PMC2704745

[b22] Rapley J. *et al.* The NIMA-family kinase Nek6 phosphorylates the kinesin Eg5 at a novel site necessary for mitotic spindle formation. J. Cell Sci. 121, 3912–3921 (2008). 19001501 10.1242/jcs.035360 PMC4066659

[b23] Bertran M. T. *et al.* Nek9 is a Plk1-activated kinase that controls early centrosome separation through Nek6/7 and Eg5. EMBO J. 30, 2634–2647 (2011). 21642957 10.1038/emboj.2011.179 PMC3155310

[b24] Sdelci S. *et al.* Nek9 phosphorylation of NEDD1/GCP-WD contributes to Plk1 control of gamma-tubulin recruitment to the mitotic centrosome. Curr. Biol. 22, 1516–1523 (2012). 22818914 10.1016/j.cub.2012.06.027

[b25] O'Regan L. *et al.* Hsp72 is targeted to the mitotic spindle by Nek6 to promote K-fiber assembly and mitotic progression. J. Cell Biol. 209, 349–358 (2015). 25940345 10.1083/jcb.201409151 PMC4427782

[b26] Belham C. *et al.* A mitotic cascade of NIMA family kinases. Nercc1/Nek9 activates the Nek6 and Nek7 kinases. J. Biol. Chem. 278, 34897–34909 (2003). 12840024 10.1074/jbc.M303663200

[b27] Rogerson D. T. *et al.* Efficient genetic encoding of phosphoserine and its nonhydrolyzable analog. Nat. Chem. Biol. 11, 496–503 (2015). 26030730 10.1038/nchembio.1823 PMC4830402

[b28] de Souza E. E. *et al.* Characterization of the human NEK7 interactome suggests catalytic and regulatory properties distinct from those of NEK6. J. Proteome Res. 13, 4074–4090 (2014). 25093993 10.1021/pr500437x PMC4156247

[b29] Gallego P., Velazquez-Campoy A., Regue L., Roig J. & Reverter D. Structural analysis of the regulation of the DYNLL/LC8 binding to Nek9 by phosphorylation. J. Biol. Chem. 288, 12283–12294 (2013). 23482567 10.1074/jbc.M113.459149 PMC3636912

[b30] Regue L. *et al.* DYNLL/LC8 protein controls signal transduction through the Nek9/Nek6 signaling module by regulating Nek6 binding to Nek9. J. Biol. Chem. 286, 18118–18129 (2011). 21454704 10.1074/jbc.M110.209080 PMC3093884

[b31] Richards M. W. *et al.* An autoinhibitory tyrosine motif in the cell-cycle-regulated Nek7 kinase is released through binding of Nek9. Mol. Cell 36, 560–570 (2009). 19941817 10.1016/j.molcel.2009.09.038 PMC2807034

[b32] Remenyi A., Good M. C., Bhattacharyya R. P. & Lim W. A. The role of docking interactions in mediating signaling input, output, and discrimination in the yeast MAPK network. Mol. Cell 20, 951–962 (2005). 16364919 10.1016/j.molcel.2005.10.030

[b33] Zhou T., Sun L., Humphreys J. & Goldsmith E. J. Docking interactions induce exposure of activation loop in the MAP kinase ERK2. Structure 14, 1011–1019 (2006). 16765894 10.1016/j.str.2006.04.006

[b34] Bastidas A. C., Wu J. & Taylor S. S. Molecular features of product release for the PKA catalytic cycle. Biochemistry 54, 2–10 (2015). 25077557 10.1021/bi500684c PMC4295794

[b35] Elling R. A., Fucini R. V. & Romanowski M. J. Structures of the wild-type and activated catalytic domains of brachydanio rerio polo-like kinase 1 (Plk1): changes in the active-site conformation and interactions with ligands. Acta Crystallogr. D Biol. Crystallogr. 64, 909–918 (2008). 18703838 10.1107/S0907444908019513

[b36] Schulze-Gahmen U., De Bondt H. L. & Kim S. H. High-resolution crystal structures of human cyclin-dependent kinase 2 with and without ATP: bound waters and natural ligand as guides for inhibitor design. J. Med. Chem. 39, 4540–4546 (1996). 8917641 10.1021/jm960402a

[b37] Lin Z., Jia L., Tomchick D. R., Luo X. & Yu H. Substrate-specific activation of the mitotic kinase Bub1 through intramolecular autophosphorylation and kinetochore targeting. Structure 22, 1616–1627 (2014). 25308863 10.1016/j.str.2014.08.020

[b38] Dodson C. A., Yeoh S., Haq T. & Bayliss R. A kinetic test characterizes kinase intramolecular and intermolecular autophosphorylation mechanisms. Sci. Signal. 6, ra54 (2013). 23821772 10.1126/scisignal.2003910

[b39] Chen J., Sawyer N. & Regan L. Protein-protein interactions: general trends in the relationship between binding affinity and interfacial buried surface area. Protein Sci. 22, 510–515 (2013). 23389845 10.1002/pro.2230 PMC3610057

[b40] Roig J., Mikhailov A., Belham C. & Avruch J. Nercc1, a mammalian NIMA-family kinase, binds the Ran GTPase and regulates mitotic progression. Genes Dev. 16, 1640–1658 (2002). 12101123 10.1101/gad.972202 PMC186374

[b41] Joshi A. *et al.* Molecular mechanisms of human IRE1 activation through dimerization and ligand binding. Oncotarget 6, 13019–13035 (2015). 25968568 10.18632/oncotarget.3864 PMC4536996

[b42] Fry A. M., Arnaud L. & Nigg E. A. Activity of the human centrosomal kinase, Nek2, depends on an unusual leucine zipper dimerization motif. J. Biol. Chem. 274, 16304–16310 (1999). 10347187 10.1074/jbc.274.23.16304

[b43] Westwood I. *et al.* Insights into the conformational variability and regulation of human Nek2 kinase. J. Mol. Biol. 386, 476–485 (2009). 19124027 10.1016/j.jmb.2008.12.033 PMC2741569

[b44] McCoy A. J. *et al.* Phaser crystallographic software. J. Appl. Crystallogr. 40, 658–674 (2007). 19461840 10.1107/S0021889807021206 PMC2483472

[b45] Adams P. D. *et al.* PHENIX: building new software for automated crystallographic structure determination. Acta Crystallogr. D Biol. Crystallogr. 58, 1948–1954 (2002). 12393927 10.1107/s0907444902016657

[b46] Emsley P. & Cowtan K. Coot: model-building tools for molecular graphics. Acta Crystallogr. D Biol. Crystallogr. 60, 2126–2132 (2004). 15572765 10.1107/S0907444904019158

[b47] Smart O. S. *et al.* Exploiting structure similarity in refinement: automated NCS and target-structure restraints in BUSTER. Acta Crystallogr. D Biol. Crystallogr. 68, 368–380 (2012). 22505257 10.1107/S0907444911056058 PMC3322596

[b48] Blanc E. *et al.* Refinement of severely incomplete structures with maximum likelihood in BUSTER-TNT. Acta Crystallogr. D Biol. Crystallogr. 60, 2210–2221 (2004). 15572774 10.1107/S0907444904016427

[b49] Chen V. B. *et al.* MolProbity: all-atom structure validation for macromolecular crystallography. Acta Crystallogr. D Biol. Crystallogr. 66, 12–21 (2010). 20057044 10.1107/S0907444909042073 PMC2803126

[b50] Krissinel E. & Henrick K. Inference of macromolecular assemblies from crystalline state. J. Mol. Biol. 372, 774–797 (2007). 17681537 10.1016/j.jmb.2007.05.022

