# Supplementary material for: Mechanistic basis of Nek7 activation through Nek9 binding and induced dimerization
Source: Nat Commun. 2015 Nov 2;6:8771. doi: 10.1038/ncomms9771 (PMC4632185; doi:10.1038/ncomms9771)
Supplement: Supplementary Information — Supplementary Figures 1-13 [file ncomms9771-s1.pdf]

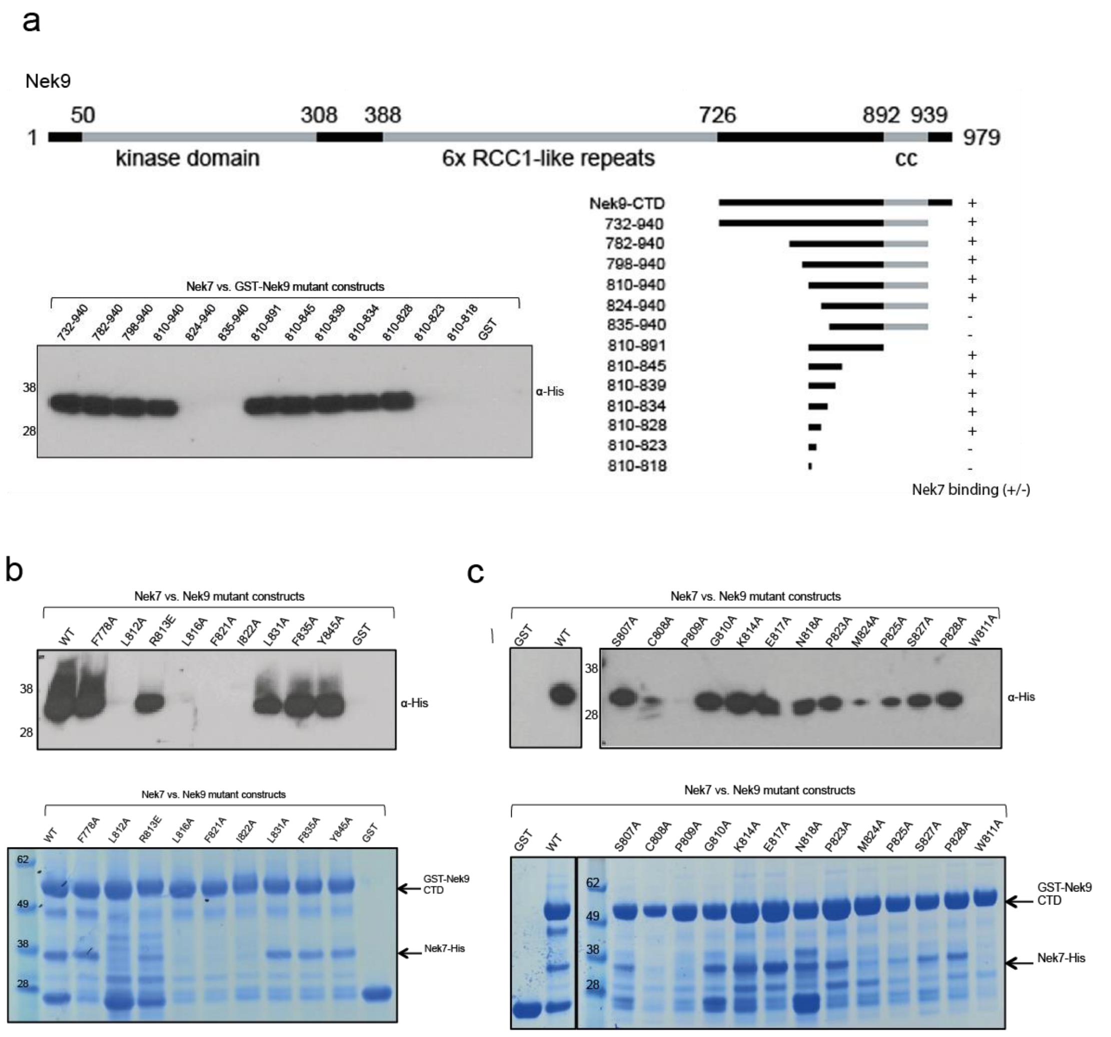

Supplementary Figure 1. Nek9 810-828 is the minimal fragment required to bind Nek7. (a) A α-His$_6$ immunoblot to show the results of a GST co-precipitation experiment to determine the binding of wild-type Nek7-His$_6$ to truncated fragments of GST-Nek9 CTD with GST as a binding control. The GST constructs used are depicted against a map of the Nek9 domain structure, with boundaries indicated by residue number. (b) *Above,* a α-His$_6$ immunoblot showing the results of a GST co-precipitation experiment to determine the binding of wild-type Nek7-His$_6$ to point mutants of the GST-Nek9 CTD. Primary antibody was 6xHis monoclonal (Clontech, Cat. #631212, 1:5000) dilution), secondary antibody was ECL Antimouse IgG conjugated to horseradish peroxidase (GE Healthcare, Cat. #NA931V, dilution 1:5000). *Below,* coomassie-stained gel. (c) *Above,* a α-His$_6$ immunoblot showing the results of a GST co-precipitation experiment to determine the binding of wild-type Nek7-His$_6$ to alanine scanning mutants within the 807-828 region of GST-Nek9 CTD. Primary antibody was 6xHis monoclonal (Clontech, Cat. #631212, 1:5000) dilution), secondary antibody was ECL Antimouse IgG conjugated to horseradish peroxidase (GE Healthcare, Cat. #NA931V, dilution 1:5000). *Below,* coomassie-stained gel.

```
Nek9_Homo_sapiens               772 PDPSGGFRGTMEADRGMEGLISPTEAMGNSNGASSSCPGWLRKELENAEF 821
Nek9_Gallus_gallus              878 PDPSGGFRGTMEADRGMGGWISTTEAKGGDSADISSCPGWLRKELENAEF 927  99%
Nek9_Meleagris_gallopavo        696 PDPSGGFRGTMEADRGMGGWISTTEAKGSDSADISSCPGWLRKELENAEF 745  92%
Nek9_Rattus_norvegicus          771 PDPSGGFRGTMEADRGMEGLISPTEAVGNSCGASSSCPGWLRKELENAEF 820  97%
Nek9_Cricetulus_griseus         594 PDPSGGFRGTMEADRGMEGLISPTEAVGNSCGASSSCPGWLRKELENAEF 643  92%
Nek9_Mus_musculus               779 PDPSGGFRGTMEADRGMEGLISPTEAVGNSCGASSSCPGWLRKELENAEF 828  92%
Nek9_Tetraodon_nigroviridis     754 ECRDRGLGGTLEDNREECFLETPMMSAANQTGD-SSCPLWLKKELEDAEF 802  82%
                                         *  *  *  *                   ************ ***

Nek9_Homo_sapiens               822 IPMP---DSPSPLSAAFSESEKDTLPYEELQGLKVASEAPLEH-KPQVEA 867
Nek9_Gallus_gallus              928 IPMP---DSPFPMSMASSEPEKETLPYQELQGLKVAPEEPTGFNKPKTEP 974
Nek9_Meleagris_gallopavo        746 IPMP---DSPFPMSMASSEPEKETLPYQELQGLKVAPEEPTGFNKPKTEP 792
Nek9_Rattus_norvegicus          821 IPMP---DSPTPLSAAFSQSEKDTLPYEELQGLKVASEVPPEH-QPAVGA 866
Nek9_Cricetulus_griseus         644 IPMP---DSPTPLSAAFSESEKDTLPYEELQGLKVASEVRPEH-QPPVGA 689
Nek9_Mus_musculus               829 IPMP---DSPAPLSAAFSQSEKDTLPYEELQGLKVASEVPPEP-QRAAGA 874
Nek9_Tetraodon_nigroviridis     803 IPMPEDGDLPTPDQLA-SHSESITLPYEELKELKAAAAA----------- 840
                                    ****   * * *   * *  *  **** **  ****
```

Supplementary Figure 2. Conservation of Nek9 in the Nek7 binding region. Multiple sequence alignment of the Nek9-CTD (Clustal W) from various species in which conservation is given in percent (%) with respect to the human sequence. Highlighted in yellow are the residues selected for mutagenesis and tested in GST co-precipitation assays for binding to Nek7 (Supplementary Figure 1). The red line indicates the region used for performing the alanine scanning GST co-precipitation experiment in Supplementary Figure 1.

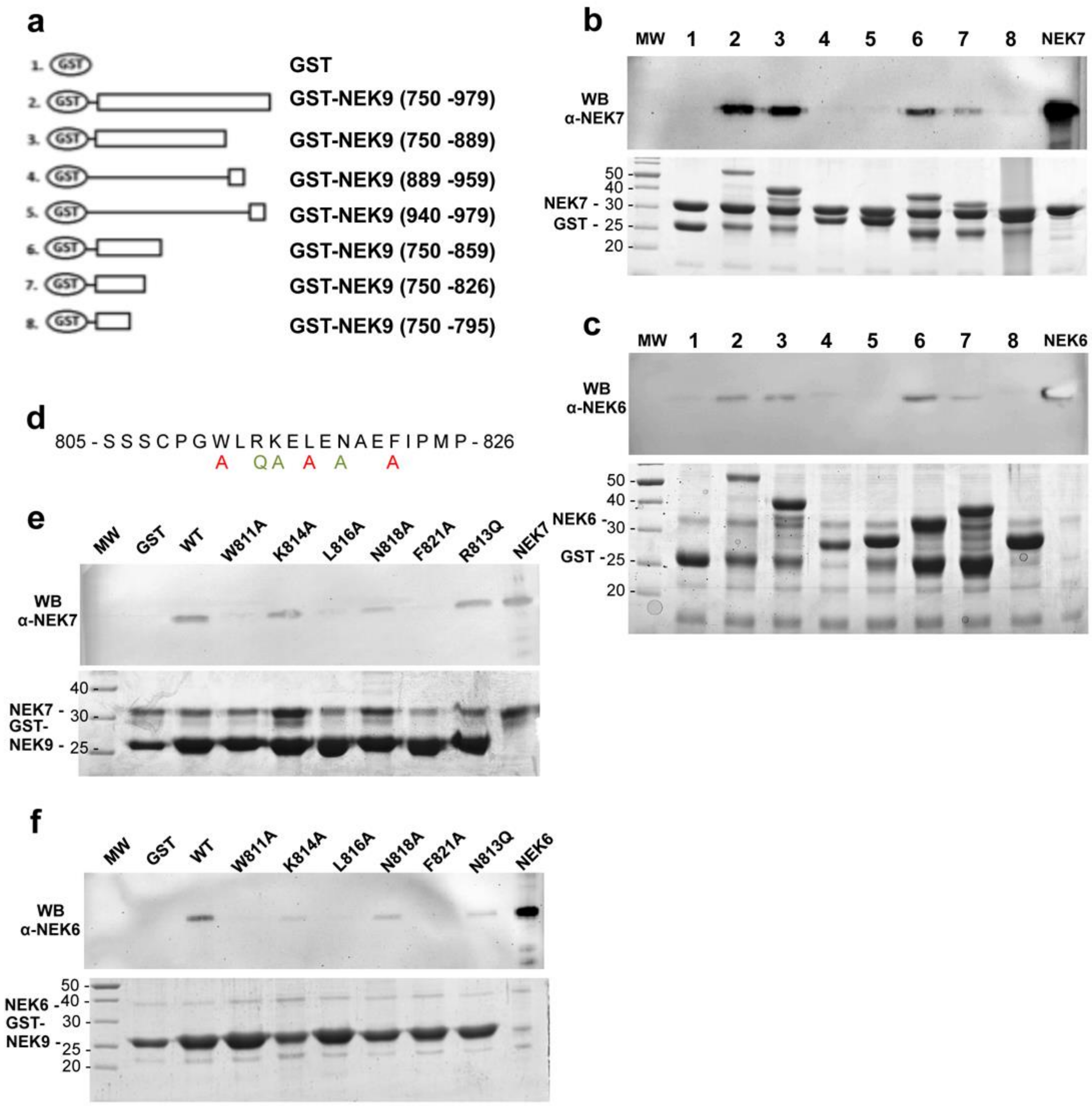

Supplementary Figure 3. Independent mapping and mutagenesis studies on the interactions between Nek9 and Nek6/Nek7. (a) Schematic representation of the GST-Nek9 C-terminal constructs tested. (b) Nek7 pull-down assays were performed using the indicated constructs. *Above*, α-Nek7 WB of GST pull-down outputs. Anti-Nek7 monoclonal antibody EPR4900 (Epitomics 3920-1) was used at a dilution of 1:10000. *Below*, Coomassie staining of input mixtures prior to pull-down. (c) Nek6 pull-downs using the indicated constructs. *Above*, α-Nek6 WB of GST pull-down outputs. Anti-Nek6 monoclonal antibody EPR5283 (Abcam ab133494) was used at a dilution of 1:1000. *Below*, Coomassie staining of input mixtures. (d) Amino acid sequence of the mapped Nek9 C-terminal Nek6 and Nek7 binding motif. *Below*, colored in red are mutations that interfered with the interaction of Nek9 with Nek6/7 and in green, mutations that did not affect the interaction in the pull-down assays. (e) Nek7 pull-downs using the indicated GST-Nek9 C-terminal constructs. *Above*, α-Nek7 WB of GST pull-down outputs. *Below*, Coomassie staining of input mixtures prior to pull-down. (f), Nek6 pull-downs using the indicated GST-Nek9 C-terminal constructs. *Above*, α-Nek6 WB of GST pull-down outputs. *Below*, Coomassie staining of input mixtures prior to pull-down.

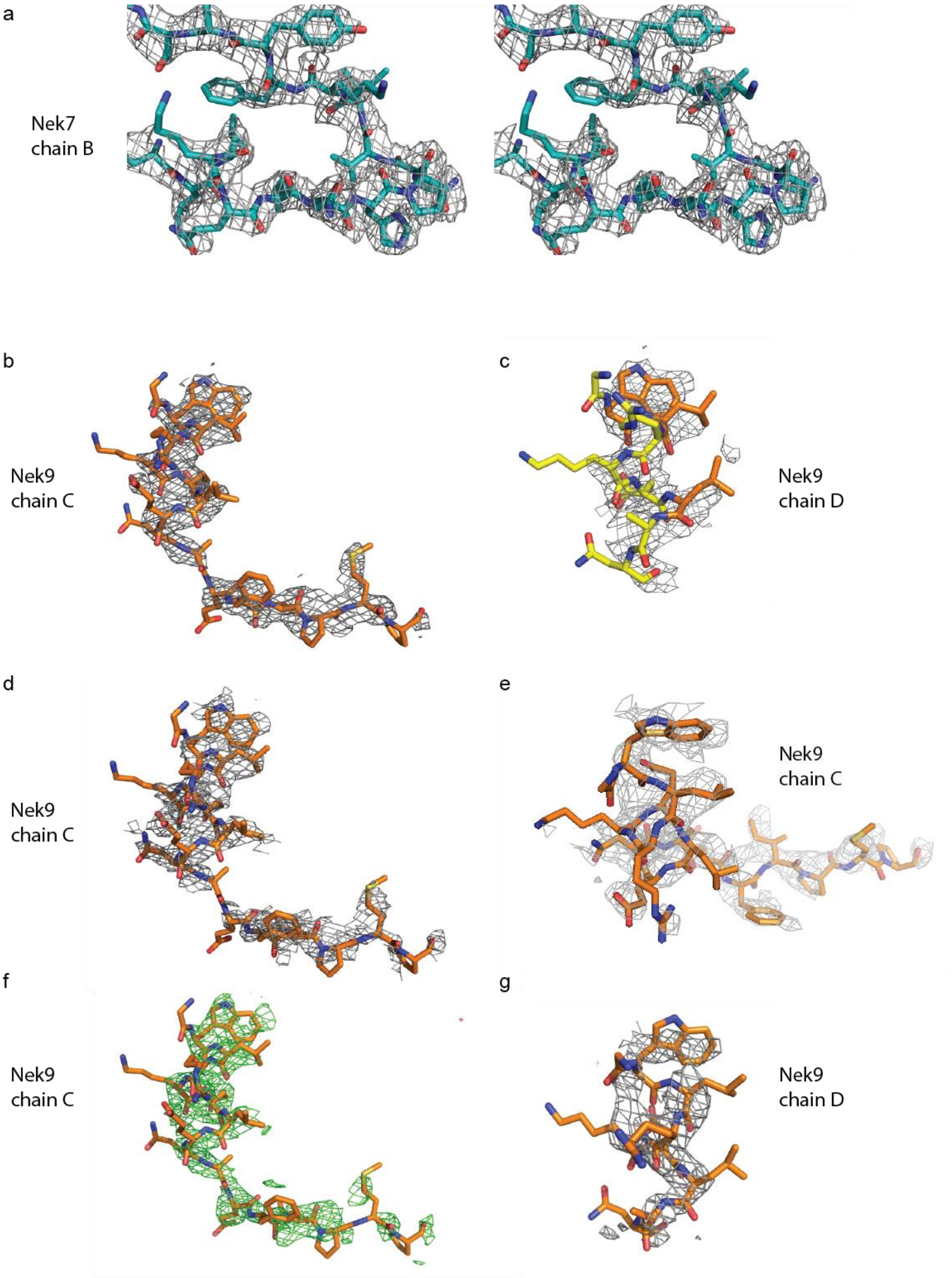

Supplementary Figure 4. Electron density maps. (a) Stereo view of the final 2Fo-Fc electron density map contoured at 1.0 σ shown as wiremesh around the Nek7 chain B in the vicinity of residue 97. (b, c) Final 2Fo-Fc electron density map contoured at 1.0 σ shown as wiremesh around the Nek9 chain C and chain D peptides. (d, e) 2Fo-Fc composite omit map with simulated annealing contoured at 0.6 σ shown as wiremesh around the Nek9 chain C. Two views are shown related by a 90° rotation around x-axis. (f) Fo-Fc composite omit map with simulated annealing contoured at 2.0 σ shown as wiremesh around the Nek9 chain C. (g) 2Fo-Fc composite omit map with simulated annealing contoured at 0.6 σ shown as wiremesh around the Nek9 chain D.

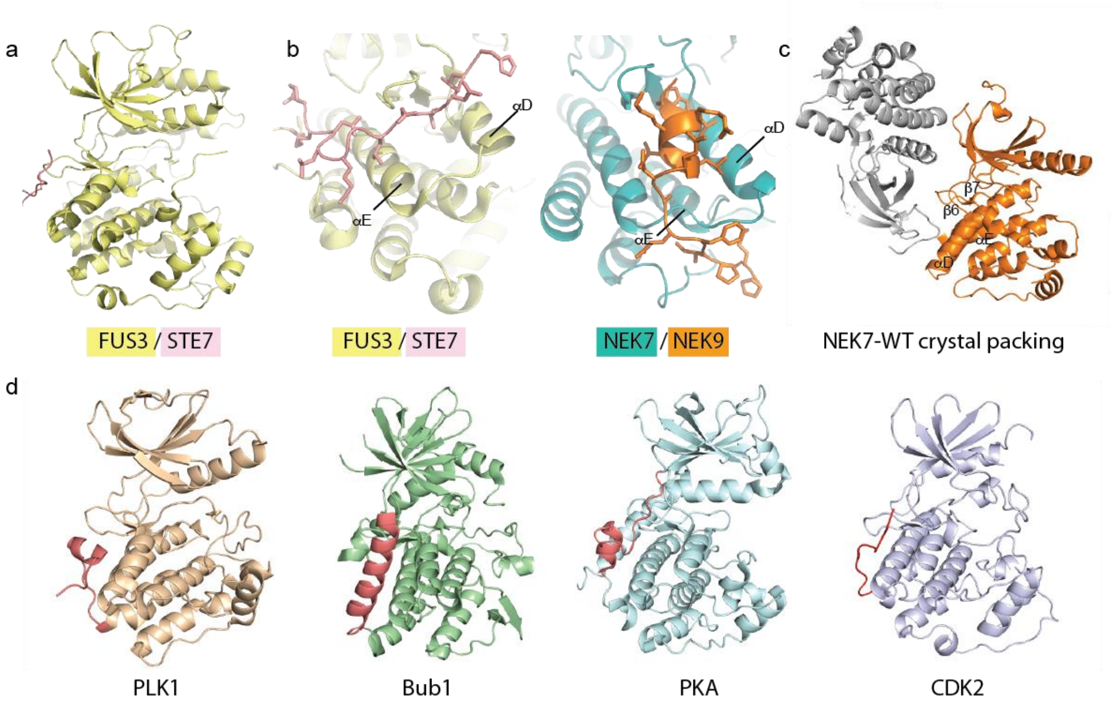

Supplementary Figure 5. Structural comparison of the Nek9 binding site on Nek7. (a) Crystal structure of FUS3 kinase (yellow) in complex with a peptide region of its binding partner STE7 (pink) based on PDB 2B9H. (b) Comparison of the STE7 (pink) binding site on FUS3 (yellow, left image) with the Nek9 (orange) binding site on Nek7 (cyan, right image). (c) Crystal structure of Nek7 alone (PDB 2WQN) showing that the Nek9 binding site is blocked by a crystal contact. (d) Crystal structures of kinases with C-terminal extensions (colored pink) that occupy the equivalent site to the Nek9 binding site on Nek7. The kinases shown are PLK1 (PDB 3D5W, wheat), Bub1 (PDB 4QPM, green), PKA (PDB 4NTS, light blue), CDK2 (PDB 1HCK, lilac).

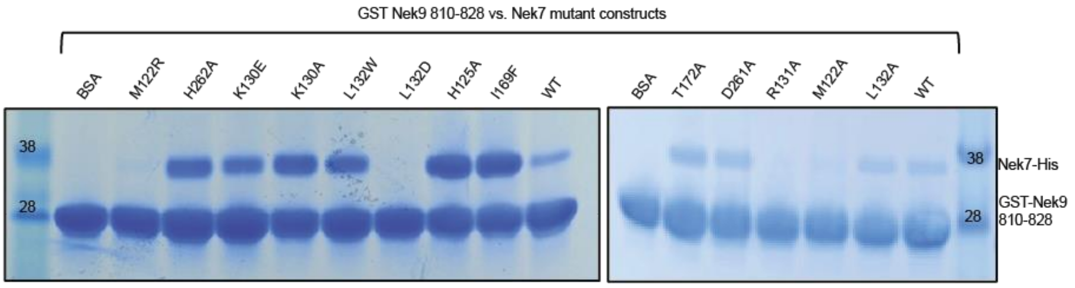

Supplementary Figure 6. Supplementary co-precipitation data. Coomassie-stained gel showing the results of a GST co-precipitation experiment testing the binding of GST Nek9 (aa810-828) to Nek7 mutants.

```
Nek6     MAGQPGHMPHGGSSNNLCHTLGPVHPPDPQRHPNTLSFRCSLADFQIEKKIGRGQFSEVY 60
Nek7     MDEQSQGMQGP-----------PVPQFQPQKALRPDMGYNTLANFRIEKKIGRGQFSEVY 49
         *  *.  *              **   :**:  ..     :**:*:**************

Nek6     KATCLLDRKTVALKKVQIFEMMDAKARQDCVKEIGLLKQLNHPNIIKYLDSFIEDNELNI 120
Nek7     RAACLLDGVPVALKKVQIFDLMDAKARADCIKEIDLLKQLNHPNVIKYYASFIEDNELNI 109
         :*:****  .*********::****** **:***.*********:***  **********

Nek6     VLELADAGDLSQMIKYFKKQKRLIPERTVWKYFVQLCSAVEHMHSRRVMHRDIKPANVFI 180
Nek7     VLELADAGDLSRMIKHFKKQKRLIPERTVWKYFVQLCSALEHMHSRRVMHRDIKPANVFI 169
         ***********:***:**********************:*********************

Nek6     TATGVVKLGDLGLGRFFSSETTAAHSLVGTPYYMSPERIHENGYNFKSDIWSLGCLLYEM 240
Nek7     TATGVVKLGDLGLGRFFSSKTTAAHSLVGTPYYMSPERIHENGYNFKSDIWSLGCLLYEM 229
         ********************:***************************************

Nek6     AALQSPFYGDKMNLFSLCQKIEQCDYPPLPGEHYSEKLRELVSMCICPDPHQRPDIGYVH 300
Nek7     AALQSPFYGDKMNLYSLCKKIEQCDYPPLPSDHYSEELRQLVNMCINPDPEKRPDVTYVY 289
         **************:***:***********.:****:**:**.*** ***.:***: **:

Nek6     QVAKQMHIWMSST-- 313
Nek7     DVAKRMHACTASSLE 304
         :***:**   :*:
```

Supplementary Figure 7. Conservation of the Nek9 binding site on Nek6. Multiple sequence alignment of full-length human Nek6 and full-length human Nek7 (ClustalW). Residues in the Nek9 binding site are highlighted in magenta, and key residues are indicated with red arrows. Residues in the back-to-back dimer interface are highlighted in blue and Tyr97 is indicated with a black arrow.

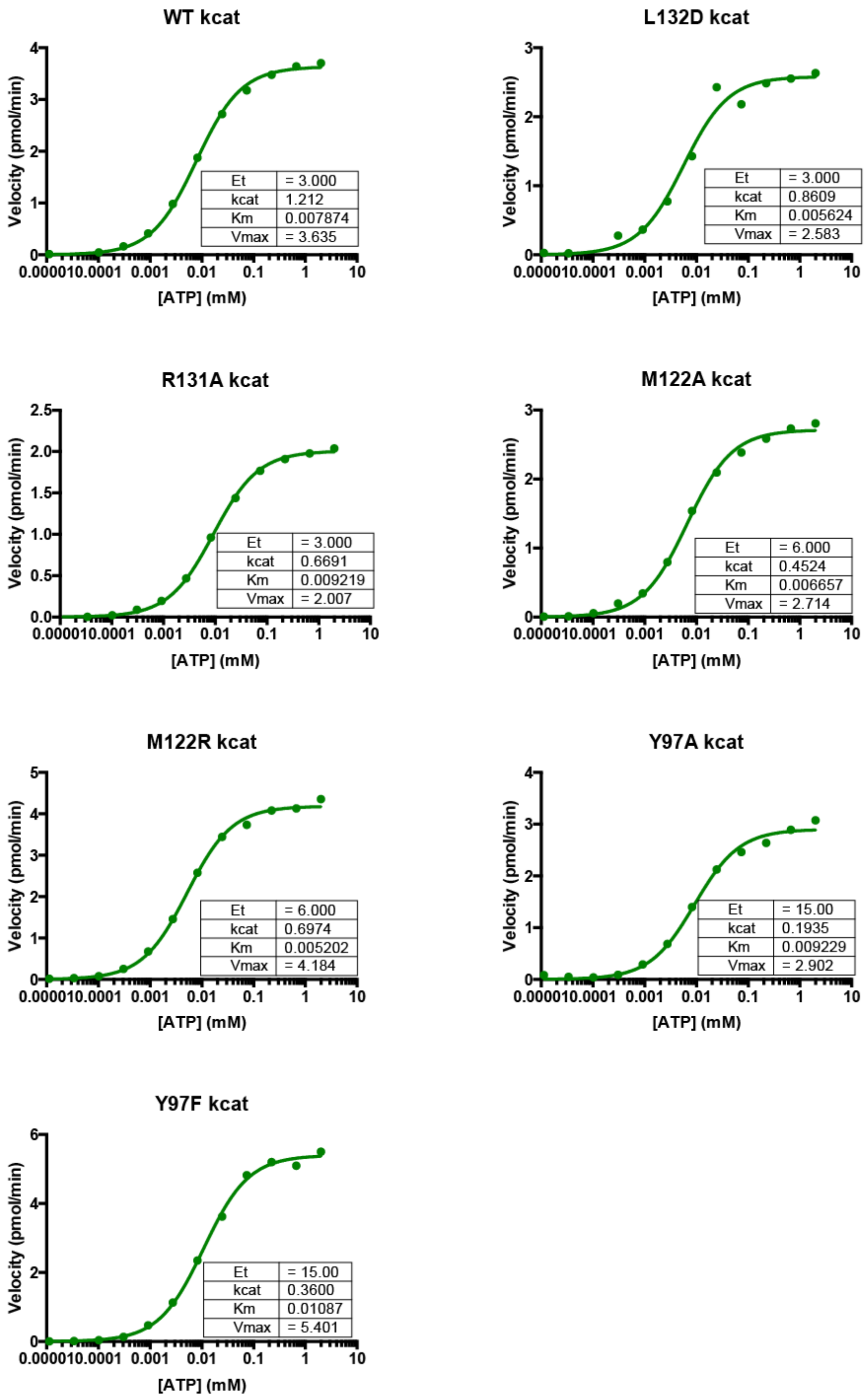

Supplementary Figure 8. Determination of the kinetic parameters of active, wild-type Nek7 and Nek7 mutants. Mobility shift assays were carried out for wild-type Nek7 and Nek7 mutants using the substrate, 5-FAM-FLAKSFGSPNRAYKK (Caliper peptide 32) and varying concentrations of ATP. K_m and k_{cat} values were calculated using the equation, Y = Et*k_{cat}*X/(K_m + X).

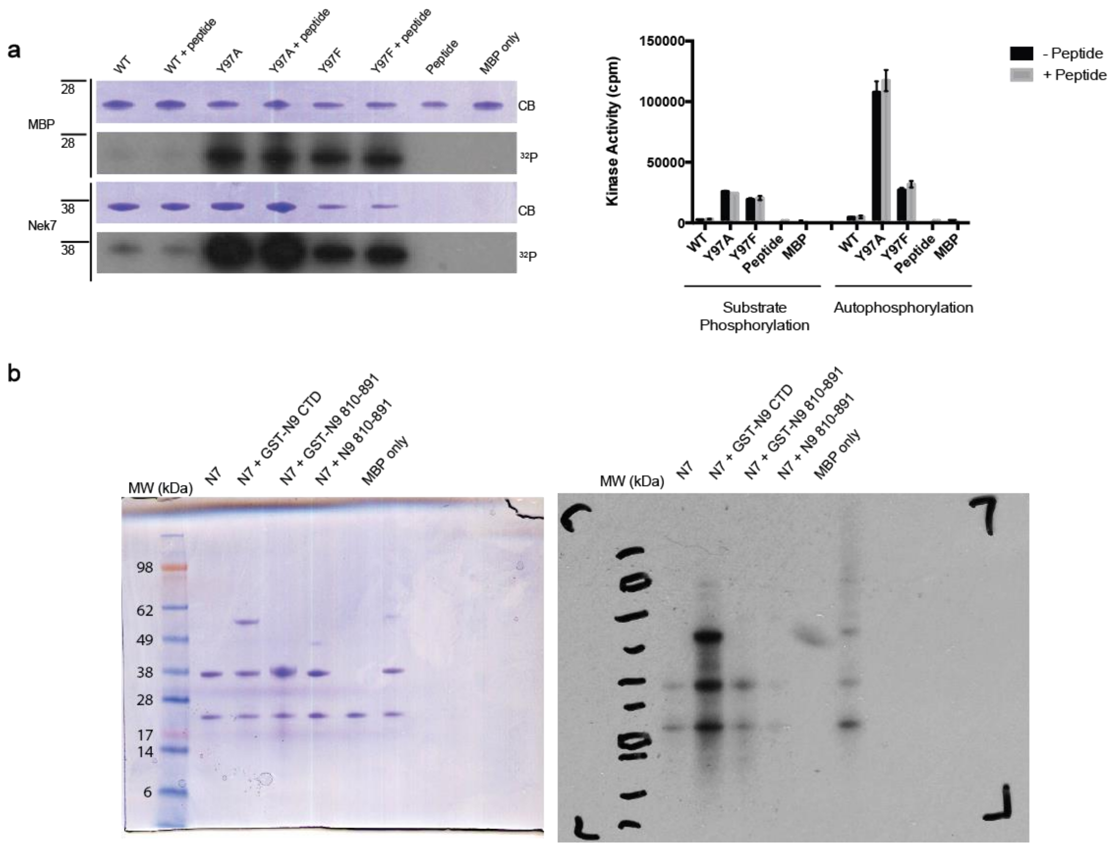

Supplementary Figure 9. Biochemical characterization of Nek7 activation by Nek9 peptide and the Nek9-CTD. (a) *In vitro* kinase activity assays show the relative activity of Nek7^{WT}, Nek7^{Y97A} and Nek7^{Y97F} with and without addition of the minimal Nek9 binding region (peptide). *Left,* Reactions were analyzed by SDS-PAGE (CB) and the resulting gel subject to autoradiography (^{32}P). *Right,* Incorporation of radioisotope was measured by scintillation counting and shown in the histogram. Error bars represent the standard deviation of two independent experiments. (b) *Left*, original coomassie-stained SDS-PAGE gel that was cropped in Figure 3a. *Right*, original autoradiograph that was cropped in Figure 3a. The additional, highly phosphorylated band at ~ 60 kDa is Nek9-CTD.

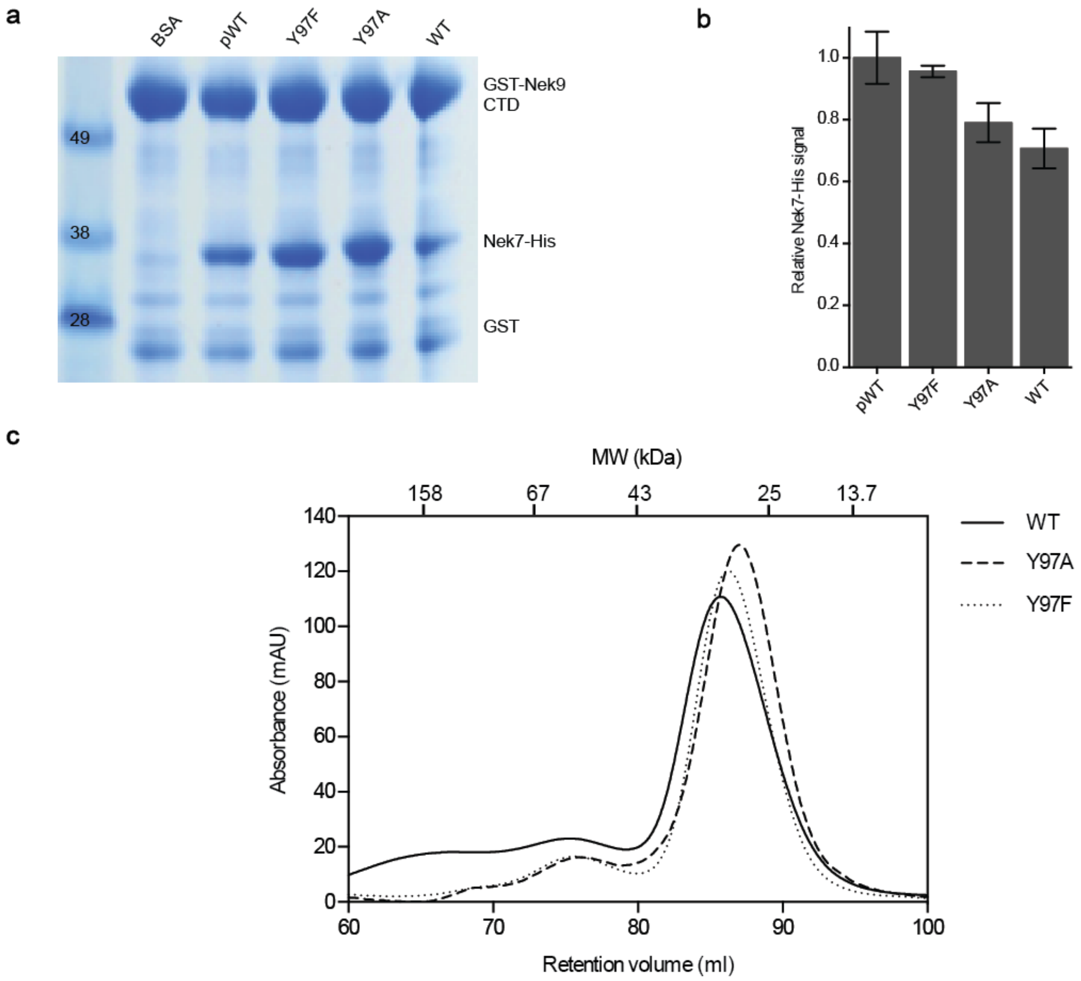

Supplementary Figure 10. Biochemical characterization of Nek7 with mutations at Tyr97. (a) Coomassie-stained gel showing the results of a GST co-precipitation experiment to test binding of GST-Nek9 CTD to Nek7^{WT}, Nek7^{Y97A}, Nek7^{Y97F} and phosphorylated Nek7^{WT} (pWT). (b) Quantification of binding between GST-Nek9 CTD and wild-type Nek7 and Nek7 mutants by immunofluorescence Western blotting, analysed as per the manufacturer's instructions (LI-COR GmbH) using an anti-His$_6$ antibody (1:1000 dilution) and IRDye 800CW secondary antibody (LI-COR GmbH). Blots were resolved using Image Studio on an Odyssey CLx Infrared imaging system (LI-COR GmbH). Statistical analysis was carried in Prism (Graphpad Software, Inc.). Error bars represent the standard error for two independent reactions. Analysis by one-way ANOVA showed mutation of Nek7 did not result in a significant change in binding between Nek7 and Nek9 CTD ($P > 0.05$). (c) Size exclusion chromatography of Nek7^{WT}, Nek7^{Y97A} and Nek7^{Y97F} on a HiLoad 16/600 Superdex 200 pg column. The column was equilibrated in 50 mM HEPES pH 7.5, 300 mM NaCl, 5% glycerol, 5 mM dithiothreitol.

a

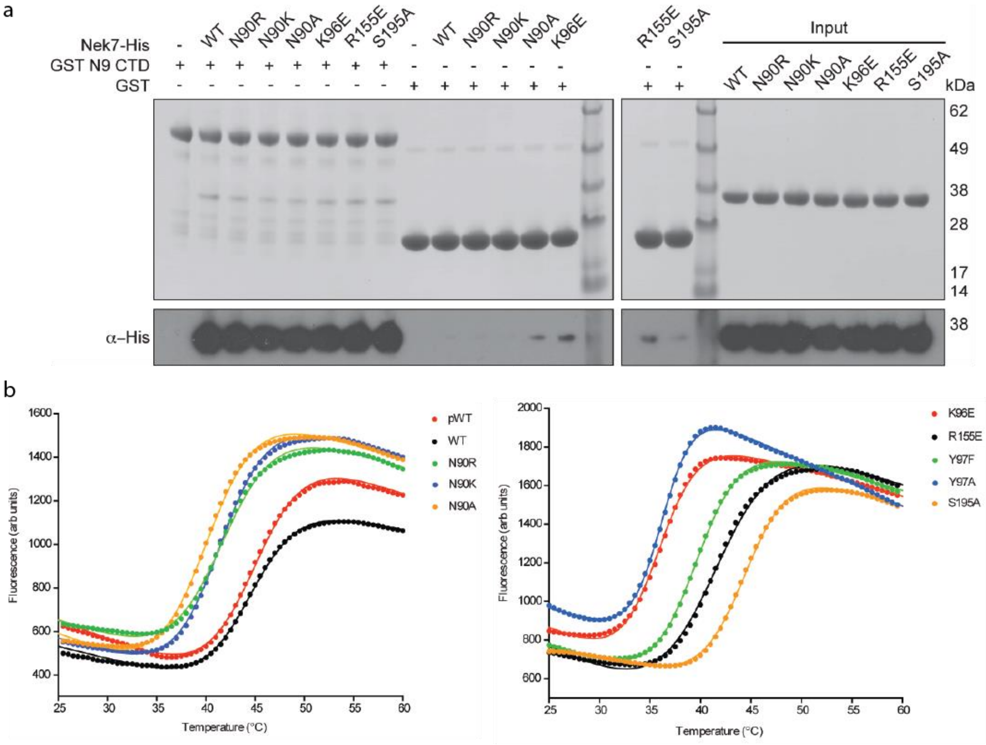

c

Protein	Apparent T_m (°C)
pWT	44.66 ± 0.13
WT	44.52 ± 0.18
N90R	41.31 ± 0.39
N90K	41.18 ± 0.26
N90A	39.91 ± 0.30
K96E	35.75 ± 0.16
R155E	41.51 ± 0.22
Y97F	39.27 ± 0.31
Y97A	36.39 ± 0.26
S195A	44.31 ± 0.15

d

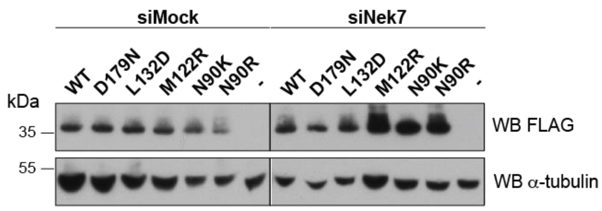

Supplementary Figure 11. Biochemical characterization of Nek7 with mutations in the back-to-back interface. (a) Co-precipitation assay between GST-Nek9 CTD and wild-type Nek7 and Nek7 mutants. GST was used as a binding control. Reactions were analysed by SDS-PAGE (top panel). Binding of Nek7 was demonstrated by Western blot using an α-His antibody (bottom panel). (b) Thermal denaturation curves for wild-type Nek7 (WT – unphosphorylated; pWT - phosphorylated) and Nek7 mutants. Proteins were heated from 4 to 99 °C in 0.5 °C increments and fluorescence readings taken. 25 μl assay samples containing 30 μM Nek7 and 1x Sypro Orange dye (Sigma) in 50 mM HEPES pH 7.5, 300 mM NaCl were heated from 4 to 99 °C in 0.5 °C increments/minute in a MyiQ Single-Colour Real-Time PCR Detection System (Biorad). Excitation and emission wavelength of 470 nm and 600 nm were used for data collection, respectively. Data were fit to a Boltzmann sigmoid function to identify the apparent melting temperature (T_m) of each sample (temperature at which 50% denaturation occurred). Eight samples were measured for each protein. (c) Apparent melting temperature (Tm, °C) from the average of eight independent reactions with standard deviation for each protein as performed in (b). (d) Western blot of cell lysates taken from each of the HeLa cell lines transfected with the Nek7-FLAG plasmid indicated (- are untransfected HeLa cells).

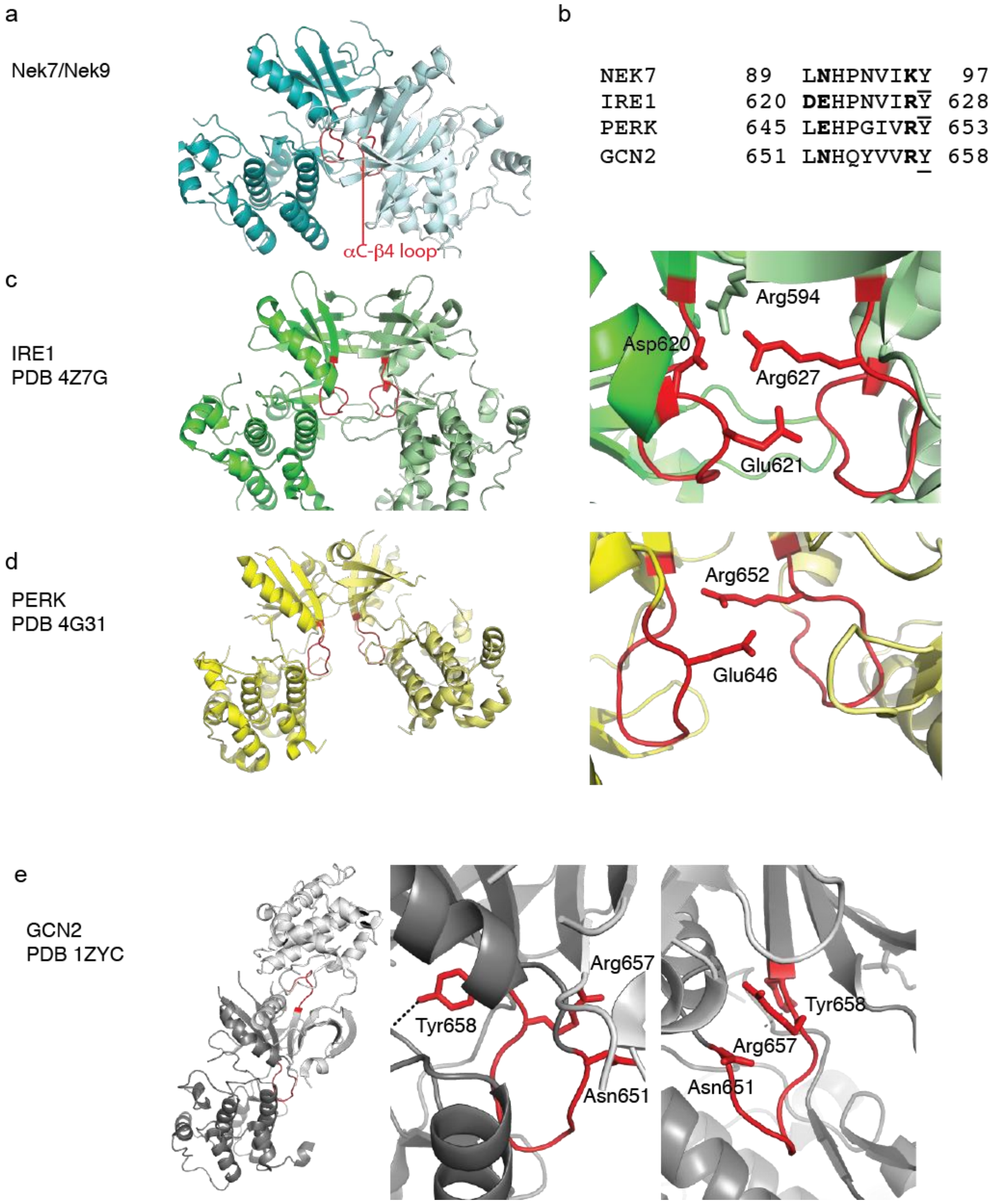

Supplementary Figure 12. Comparison of kinase back-to-back dimers. (a, c-e) *Left*, Nek7/Nek9, IRE1, PERK and GCN2 back-to-back dimers are shown in cartoon representation. In each case, one protomer was aligned with Nek7 (colored more intensely). The αC-β4 loop is colored red. Note the distinctive back-to-back GCN2 dimer in which the two protomers are oriented at an angle of 180°. (b) Sequence alignment of αC-β4 loops, with key residues in the back-to-back interface highlighted in bold, and the conserved tyrosine that is implicated in auto-inhibition underlined. (c,d) *Right,* magnified view of the IRE1 and PERK back-to-back interfaces showing the salt-bridges. (e) *Centre,* GCN2 is in a Y-down conformation and the side chain of Tyr658, equivalent to Tyr97 of Nek7, forms a H-bond with the DFG-motif. *Right,* Arg657 of GCN2, equivalent to Lys96 of Nek7, obscures the main chain of Tyr658. This is similar to the situation in the Tyr-down auto-inhibited conformation of Nek7.

a

```
NEK1     YVRLQKIGEGSFGKAILVKST---EDGRQYVIKEIN-ISRMSSKEREESRREVA------
NEK2     YEVLYTIGTGSYGRCQKIRRK---SDGKILVWKELD-YGSMTEAEKQMLVSEVN------
NEK3     YMVLRMIGEGSFGRALLVQHE---SSNQMFAMKEIR-LPKSFSN-TQNSRKEAV------
NEK4     YCYLRVVGKGSYGEVTLVKHR---RDGKQYVIKKLN-LRNASSRERRAAEQEAQ------
NEK5     YDVIKAIGQGAFGKAYLAKGK---SDSKHCVIKEIN-FEKMPIQEKEASKKEVI------
NEK6     FQIEKKIGRGQFSEVYKATCL---LDRKTVALKKVQIFEMMDAKARQDCVKEIG------
NEK7     FRIEKKIGRGQFSEVYRAACL---LDGVPVALKKVQIFDLMDAKARADCIKEID------
NEK8     YERIRVVGRGAFGIVHLCLRK---ADQKLVIIKQIP-VEQMTKEERQAAQNECQ------
NEK9     YIPIRVLGRGAFGEATLYRRT---EDDSLVVWKEVD-LTRLSEKERRDALNEIV------
NEK10    YAILDHLGSGAFGCVYKVRKH---SGQNLLAMKEVNLHNPAFGKDKKDRDSSVRNIVSEL
NEK11    YVLQQKLGSGSFGTVYLVSDKKAKRGEELKVLKEIS-VGELNPNETVQANLEAQ------
cons     :     :* * :.            .      *::                .

NEK1     --VLANMKHPNIVQYRESFEENG--SLYIVMDYCEGGDLFKRINA--QKGVLFQEDQILD
NEK2     --LLRELKHPNIVRYYDRIIDRTNTTLYIVMEYCEGGDLASVITKGTKERQYLDEEFVLR
NEK3     --LLAKMKHPNIVAFKESFEAEG--HLYIVMEYCDGGDLMQKIKQ--QKGKLFPEDMILN
NEK4     --LLSQLKHPNIVTYKESWEGGDG-LLYIVMGFCEGGDLYRKLKE--QKGQLLPENQVVE
NEK5     --LLEKMKHPNIVAFFNSFQENG--RLFIVMEYCDGGDLMKRINR--QRGVLFSEDQILG
NEK6     --LLKQLNHPNIIKYLDSFIEDN--ELNIVLELADAGDLSQMIKYFKKQKRLIPERTVWK
NEK7     --LLKQLNHPNVIKYYASFIEDN--ELNIVLELADAGDLSRMIKHFKKQKRLIPERTVWK
NEK8     --VLKLLNHPNVIEYYENFLEDK--ALMIAMEYAPGGTLAEFIQK--RCNSLLEEETILH
NEK9     --ILALLQHDNIIAYYNHFMDNT--TLLIELEYCNGGNLYDKILR--QKDKLFEEEMVVW
NEK10    TIIKEQLYHPNIVRYYKTFLEND--RLYIVMELIEGAPLGEHFSSLKEKHHHFTEERLWK
NEK11    --LLSKLDHPAIVKFHASFVEQD--NFCIITEYCEGRDLDDKIQEYKQAGKIFPENQIIE
cons       :   : *  :: :           : *      .  *   :    .    : *  :

NEK1     WFVQICLALKHVHDR-----KILHRDIKSQNIFLTKDGTVQ-LGDFGIARVLNSTVELAR
NEK2     VMTQLTLALKECHRRSDGGHTVLHRDLKPANVFLDGKQNVK-LGDFGLARILNHDTSFAK
NEK3     WFTQMCLGVNHIHKK-----RVLHRDIKSKNIFLTQNGKVK-LGDFGSARLLSNPMAFAC
NEK4     WFVQIAMALQYLHEK-----HILHRDLKTQNVFLTRTNIIK-VGDLGIARVLENHCDMAS
NEK5     WFVQISLGLKHIHDR-----KILHRDIKAQNIFLSKNGMVAKLGDFGIARVLNNSMELAR
NEK6     YFVQLCSAVEHMHSR-----RVMHRDIKPANVFITATGVVK-LGDLGLGRFFSSETTAAH
NEK7     YFVQLCSALEHMHSR-----RVMHRDIKPANVFITATGVVK-LGDLGLGRFFSSKTTAAH
NEK8     FFVQILLALHHVHTH-----LILHRDLKTQNILLDKHRMVVKIGDFGISKILSSKS----
NEK9     YLFQIVSAVSCIHKA-----GILHRDIKTLNIFLTKAN-LIKLGDYGLAKKLNSEYSMAE
NEK10    IFIQLCLALRYLHKE----KRIVHRDQTPNNIMLGDKDKVT-VTDFGLAKQKQENSKLTS
NEK11    WFIQLLLGVDYMHER-----RILHRDLKSKNVFLKN-NLLK-IGDFGVSRLLMGSCDLAT
cons      : *:  .:   *        ::*** .. *:::     :  : * * .:

NEK1     TCIGTPYYLSPEICENKPYNNKSDIWALGCVLYELCTLKHAFEAG--SMKNLVLKIISGS
NEK2     TFVGTPYYMSPEQMNRMSYNEKSDIWSLGCLLYELCALMPPFTAF--SQKELAGKIREGK
NEK3     TYVGTPYYVPPEIWENLPYNNKSDIWSLGCILYELCTLKHPFQAN--SWKNLILKVCQGC
NEK4     TLIGTPYYMSPELFSNKPYNYKSDVWALGCCVYEMATLKHAFNAK--DMNSLVYRIIEGK
NEK5     TCIGTPYYLSPEICQNKPYNNKTDIWSLGCVLYELCTLKHPFEGN--NLQQLVLKICQAH
NEK6     SLVGTPYYMSPERIHENGYNFKSDIWSLGCLLYEMAALQSPFYGDKMNLFSLCQKIEQCD
NEK7     SLVGTPYYMSPERIHENGYNFKSDIWSLGCLLYEMAALQSPFYGDKMNLYSLCKKIEQCD
NEK8     ----TPCYISPELCEGKPYNQKSDIWALGCVLYELASLKRAFEAA--NLPALVLKIMSGT
NEK9     TLVGTPYYMSPELCQGVKYNFKSDIWAVGCVIFELLTLKRTFDAT--NPLNLCVKIVQGI
NEK10    -VVGTILYSCPEVLKSEPYGEKADVWAVGCILYQMATLSPPFYST--NMLSLATKIVEAV
NEK11    TLTGTPHYMSPEALKHQGYDTKSDIWSLACILYEMCCMNHAFAGS--NFLSIVLKIVEGD
cons         *  *  **      *. *:*:*::.* ::::  :  .* .   .   :  :: .

NEK1     --FPPVS-LHYSYDLRSLVSQLFKRNPRDRPSVNSILEKGFI
NEK2     --FRRIP-YRYSDELNEIITRMLNLKDYHRPSVEEILENPLI
NEK3     --ISPLP-SHYSYELQFLVKQMFKRNPSHRPSATTLLSRGIV
NEK4     --LPAMP-RDYSPELAELIRTMLSKRPEERPSVRSILRQPYI
NEK5     --FAPIS-PGFSRELHSLISQLFQVSPRDRPSINSILKRPFL
NEK6     --YPPLPGEHYSEKLRELVSMCICPDPHQRPGSLEQLRPQGS
NEK7     --YPPLPSDHYSEELRQLVNMCINPDPEKRPDV---------
NEK8     --FAPIS-DRYSPELRQLVLSLLSLEPAQRPPLSHIMAQPLC
NEK9     RAMEVDS-SQYSLELIQMVHSCLDQDPEQRPTADELLDRPLL
NEK10    --YEPVPEGIYSEKVTDTISRCLTPDAEARPDIVEVSSMISD
NEK11    --TPSLP-ERYPKELNAIMESMLNKNPSLRPSAIEILKIPYL
cons           .   :. .:   :   :      **
```

b

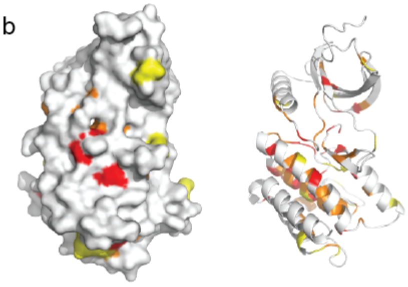

c

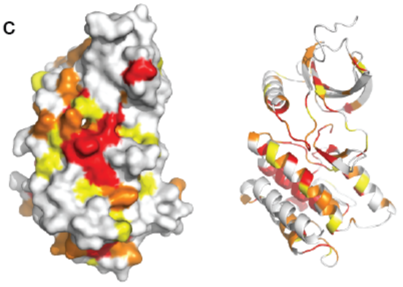

Supplementary Figure 13. Sequence conservation of human Nek kinases. (a) Sequence alignment of human Nek kinase catalytic domains. The αC-β4 loop sequences are enclosed in a rectangle and the auto-inhibitory tyrosine residue is marked with a red arrow. (b) Structure of Nek7 viewed at the back-to-back interface – surface (left) and cartoon (right). Residues are colored by sequence conservation among all 11 human Neks – identical (red), strong conservation (orange), weak conservation (yellow) and not conserved (white). (c) As in (b) with residues colored by conservation between human Neks 2,6,7,8. This subgroup of Neks has the most similar sequences in the αC-β4 loop.
